# *Lactobacillus fermentum* ZS40 Ameliorates Inflammation in Mice With Ulcerative Colitis Induced by Dextran Sulfate Sodium

**DOI:** 10.3389/fphar.2021.700217

**Published:** 2021-11-19

**Authors:** Zixia Chen, Long Yi, Yanni Pan, Xingyao Long, Jianfei Mu, Ruokun Yi, Xin Zhao

**Affiliations:** ^1^ Chongqing Collaborative Innovation Center for Functional Food, Chongqing Engineering Research Center of Functional Food, Chongqing Engineering Laboratory for Research and Development of Functional Food, Chongqing University of Education, Chongqing, China; ^2^ Chongqing Key Laboratory of Translational Research for Cancer Metastasis and Individualized Treatment, Chongqing University Cancer Hospital, Chongqing, China

**Keywords:** *Lactobacillus fermentum*, DSS, ulcerative colitis, NF-κB, MAPK

## Abstract

Ulcerative colitis is an inflammatory disease of the intestine caused by many reasons, and it may even develop into colon cancer. Probiotics are normal bacteria that exist in the human body and have been proven to regulate the balance of intestinal flora and alleviate inflammation. The current study aimed to study the effect of *Lactobacillus fermentum* ZS40 (ZS40) on dextran sulfate sodium (DSS)-induced ulcerative colitis mice. The length and weight of the colon were measured, and the histopathological morphological changes of colon tissue were observed to evaluate the effects of ZS40 on colitis. Biochemical kits, ELISA kits, real-time quantitative PCR (RT-qPCR), and western blot were also used to detect the effects of ZS40 on serum and colon tissue related oxidative indicators and pro-inflammatory and anti-inflammatory cytokines. We found that ZS40 could reduce colonic inflammatory cell infiltration and goblet cell necrosis, increase total superoxide dismutase and catalase in mouse serum, and reduce myeloperoxidase and malondialdehyde levels. ZS40 could down-regulate the level of proinflammatory cytokines and up-regulate the level of anti-inflammatory cytokines. More importantly, ZS40 down-regulated the relative expression of nuclear factor-κB p65 (NF-κBp65), IL-6, and TNF-α mRNA and protein, up-regulated the relative expression of inhibitor kapa B alpha (IκB-α). By regulating the NF-κB and MAPK pathways to down-regulated the relative expression of p38 and JNK1/2 mRNA and p38, p-p38, JNK1/2, and p-JNK1/2 proteins. Our study suggested that ZS40 may serve as a potential therapeutical strategy for ulcerative colitis.

## Introduction

Ulcerative colitis (UC) is a chronic nospecific inflammatory bowel disease. The main symptoms include abdominal pain, diarrhea, blood in the stool, and weight loss, etc (Baumgart and Carding, 2007). The disease has varying levels of severity and can easily relapse, which seriously affects the quality of life of patients and further develop into colon cancer (Burisch and Munkholm, 2015). It occurs worldwide, but the incidence varies by race, region, age, and gender (Ng et al., 2017). Its pathogenesis may be related to the comprehensive effects of multiple factors and multiple links, including immune disorder, intestinal flora imbalance, immune response imbalance, and other factors (Sun et al., 2019). Epidemiological data have shown that the incidence and prevalence of ulcerative colitis exhibit a rising trend, which may be attributable to changes in patients’ lifestyle, diet, and routines (da Silva et al., 2014). Common drugs for the treatment of colitis include sulfasalazine, aminosalicylic acid, and glucocorticoids. However, the continuous use of drugs can exert certain side effects on the human body (Pay et al., 2000; Loftus et al., 2004). Therefore, highly safe treatment for ulcerative colitis and one with few side effects has drawn scientific interest in recent years.

Xinjiang, is one of the places in northwestern China where ethnic minorities gather. Dairy products are part of traditional food culture and thus is indispensable. Compared with modern techniques, the traditional approach to dairy production is more ecological and natural, and the types of probiotics produced are more abundant. Dairy products provide a rich resource of lactic acid bacteria (Colombo et al., 2018). Screening of probiotic lactic acid bacteria from traditional dairy products not only enriches the *Lactobacillus* strain library, but also provides raw materials for beneficial strains in industrial production.

Probiotics are active microorganisms that exist in the human gastrointestinal tract and are beneficial to human health (Cordonnier et al., 2015). They can perform various physiological and biochemical functions in the human body, including maintaining the balance of intestinal flora, improving the function of intestinal mucosa, inhibiting the growth of intestinal pathogenic bacteria, and preventing gastrointestinal infections and inflammatory bowel diseases (Bezirtzoglou and Stavropoulou, 2011). Lactic acid bacteria are considered to be the safest probiotics. Many studies have examined the efficacy of lactic acid bacteria. Woo et al. evaluated the benefit of the plant-type *Lactobacillus pentosus* (*L. pentosus*) C29 to memory impairment induced by d-galactose in aging mice. The result indicated that treatment with plant-type *L. pentosus* C29 could delay memory decline induced by d-galactose (Woo et al., 2014). Long et al. evaluated the effect of *L. plantarum* KFY04 in the peroxisome proliferators-activated receptors (PPARs) pathway on obese mice. The results showed that *L. plantarum* KFY04 could inhibit obesity in mice and reduce oxidative damage and inflammation (Long et al., 2020). Hu et al. investigated the alleviating effect of *L. plantarum* LP33 on liver injury in rats with Pb poisoning. The results showed that *L. plantarum* LP33 could reduce Pb-induced oxidative stress and inflammation in the liver of rats and increase Pb excretion in rats with Pb poisoning (Hu et al., 2020). Therefore, it is very meaningful to screen lactic acid strains with special effects and apply them in production.

In the current study, we isolated and purified ZS40 from traditionally fermented yak yogurt in Paleksu Kaisk grassland in Zhaosu County, Xinjiang, China. We used 3% DSS (MP Biomedical, Santa Ana, CA) to induce mice to establish the model of ulcerative colitis. Moreover, we conducted a preliminary analysis of the mechanism of action by measuring the length and weight of colon tissues, observing pathological changes, measuring the levels of related inflammatory factors in the serum, and detecting the relative expression levels of related mRNA and protein indicatorsin the colon tissues. Our results provide a theoretical basis for further research on the treatment of ulcerative colitis by lactic acid bacteria and the development of new lactic acid bacteria preparations.

## Materials and Methods

### Experimental Strain

ZS40 was isolated from traditionally fermented yak yogurt in the Paleksu Kaisk grassland in Zhaosu County. This strain was identified using the Basic Partial Comparison Search Tool or BLAST of the National Center for Biotechnology Information. It was deposited with China General Microbial Culture Collection Center (CGMCC, Beijing, China; CGMCC No: 18226). *Lactobacillus bulgaricus* (LB; AB200048) was purchased from China Center for Type Culture Collection (CCTCC) (Wuhan, Hubei, China) and used as a positive control.

### Testing for Tolerance to pH 3.0 Artificial Gastric Juice

The artificial gastric juice (0.2% NaCl+0.35% pepsin, Beijing Solarbio Science and Technology Co. Ltd., Beijing, China) was adjusted to pH 3.0 with 1 mol/L HCl and then filtered and sterilized with a 0.22 μm filter. ZS40 was cultured twice in 5 ml of MRS medium to activate the strain, centrifuged at 3,000 rpm for 10 min to collect the bacterial pellet, washed twice with sterile normal saline, and resuspended in 5 ml of sterile normal saline. The bacterial solution was mixed with sterile artificial gastric juice (1:9 v/v), incubated at 37°C. The survival rates at 0 and 3 h were calculated using the following formula (Mu et al., 2020):

$$survival\;rate\left(\%\right)=\frac{3h\;viable\;count\left(CFU\;mL^{-1}\right)}{0h\;viable\;count\left(CFU\;mL^{-1}\right)}\times 100$$

### Determination of the 0.3% Bile Salt Growth Rate

The activated ZS40 was inoculated into an MRS medium supplemented with 0.2% sodium thioglycolate (MRS–THIO) medium with bile salt contents of 0.0 and 0.3% at 2% inoculum. After the bacteria were cultured with the blank MRS–THIO medium at 37°C for 24 h as a control, the blank and inoculation media were separately loaded into 96-well plates (200 μl/well), and the OD_600nm_ values of different concentrations of the medium were determined. The growth efficiency was calculated as follows (Mu et al., 2020):

$$growth\;efficiency\left(\%\right)=\frac{OD_{600}\;of\;0.3\%\;bile\;salt\;medium-blank\;medium}{OD_{600}\;of\;0.0\%\;bile\;salt\;medium-blank\;medium}\times 100$$

### Animal Model Establishment

A total of 50 male SPF C57BL/6J mice aged 7 weeks were purchased from the Experimental Animal Center of Chongqing Medical University, (SCXK (YU) 2017–0001). After adaptive feeding for 7 d, the mice were randomly divided into five groups: the control group, the DSS group, the sulfasalazine group (sulfasalazine, SSZ, 500 mg/kg, Macklin, China), the ZS40 group (ZS40, 1.0 × 10^9^ CFU/kg), and the *L. bulgaricus* group (LB, 1.0 × 10^9^ CFU/kg). The specific treatment methods were as follows: a. Throughout the experiment, the control group received standard food and drinking water as well as daily gavage of normal saline 0.1 ml/10 g (mouse body weight); b. The DSS group received standard food and drinking water, the drinking water was replaced with a 3% DSS solution in the third week of the experiment, and 0.1 ml/10 g of normal saline (mouse body weight) was administered daily during the experiment; c. The mice in the SSZ, ZS40, and LB groups were administered with corresponding samples by gavage daily. The drinking water was replaced with a solution containing 3% DSS in the third week of the experiment. During the experiment, the mice were gavage at a specific time, and their body weights were recorded. The experiment ended in the fifth week.

### Sample Collection

After 5 weeks, the mice were sacrificed by cervical dislocation. Blood from the eyeball was collected into a centrifuge tube and then centrifuged at 4°C for 10 min at 4,000 rpm. The upper layer of the serum was collected then aliquoted into a 200 μl centrifuge tube and preserved in an ultra-low-temperature refrigerator set to −80°C (Thermo Fisher Scientific Co. Ltd., Shanghai, China). After blood collection, the mice were dissected, and the colon was taken out to measure its length and weight. The colon was photographed for recording. A colon tissue sample measuring 0.5 cm was sliced and then fixed in 10% formalin solution. The remaining colon was frozen in liquid nitrogen and placed in an ultralow-temperature refrigerator set to −80°C for subsequent use.

### Histological Observation

Hematoxylin–eosin (HE) staining of the colon tissue was conducted. The staining process included fixing, embedding, sectioning, pressing, dewaxing, staining, and fixing. The colon morphology was evaluated based on degree of inflammation, crypt damage, and mucosal damage. The pathological form of the colon tissue was observed using an upright microscope (Olympus Scientific Instruments Co. Ltd., Guangdong, China).

### Determination of T-SOD, MPO, CAT, and MDA in Serum

The collected plasma was centrifuged at 4°C and 4,000 rpm for 10 min, and the upper layer was removed. The activity levels of total superoxide dismutase (T-SOD; kit number A007-1–1), myeloperoxidase (MPO; A044), and catalase (CAT; A003-1–2), as well as the malondialdehyde (MDA; A001-1–2) content in mice serum were determined following the instructions in the biochemical kit (Nanjing Jiancheng Bioengineering Institute, Nanjing, China).

### Determination of IL-1β, IL-6, IL-10, and TNF-α Levels in Serum

The collected plasma was centrifuged at 4°C and 4,000 rpm for 10 min, and then the serum in the upper layer was removed. The interleukin-1β (IL-1β), interleukin-6 (IL-6), interleukin-10 (IL-10), and fumor necrosis factor (TNF-α) cytokine levels in the mice serum were determined in accordance with the instruction provided in the enzyme-linked immunoabsorbent (ELISA) kit (Nanjing Jiancheng Bioengineering Institute).

### Real-Time Fluorescence-Based Quantitative PCR Determination of the Mouse Liver

Total RNA was extracted with TRIzol Reagent (Thermo Fisher Scientific Inc., Waltham, MA, United States), and reverse transcription of RNA into cDNA was performed following the instructions provided in the Revert Aid First Strand cDNA Synthesis Kit (Thermo Fisher Scientific Inc.). The microphotometer (Hangzhou Aosheng Instrument Co. Ltd., Hangzhou, China) was used to determine the concentration and purity of RNA and cDNA. Amplification was performed with a real-time polymerase chain reaction machine (Thermo Fisher Scientific Inc.) under the following conditions for 40 cycles: 95°C for 15 s, 60°C for 30 s, 95°C for 35 s. β-actin was used as the internal reference gene, and the expression of each gene was calculated using the 2^−ΔΔCT^ method (Wang et al., 2019). The primer sequences used in this study is shown in Table 1.

**TABLE 1:** Sequences of primers used in the qPCR assay.

Gene name	Sequence
β-actin	Forward: 5′- ATG​GAG​CCG​GAC​AGA​AAA​GC-3′
	Reverse: 5′- TGG​GAG​GTG​TCA​ACA​TCT​TCT​T-3′
NF-κB	Forward: 5′- ATG​GCA​GAC​GAT​GAT​CCC​TAC-3′
	Reverse: 5′- CGG​AAT​CGA​AAT​CCC​CTC​TGT​T-3′
IκB-α	Forward: 5′- TGA​AGG​ACG​AGG​AGT​ACG​AGC-3′
	Reverse: 5′- TGC​AGG​AAC​GAG​TCT​CCG​T -3′
TNF-α	Forward: 5′- CTG​AAC​TTC​GGG​GTG​ATC​GG -3′
	Reverse: 5′- GGC​TTG​TCA​CTC​GAA​TTT​TGA​GA -3′
IL-6	Forward: 5′- CTG​CAA​GAG​ACT​TCC​ATC​CAG -3′
	Reverse: 5′- AGT​GGT​ATA​GAC​AGG​TCT​GTT​GG -3′
p38	Forward: 5′- ACCTAGCTGTGAACGA -3′
	Reverse: 5′- GTA​GCC​ACG​TAG​CCT​GTC​ATC -3′
JNK1/2	Forward: 5′- TGG​ACT​TGG​AGG​AGA​GAA​CC -3′
	Reverse: 5′- CAT​TGA​CAG​ACG​ACG​ATG​ATG -3′

NF-κBp65 = nuclear factor-κB p65, IκB-α = nuclear factor of κ-light polypeptide gene enhancer in B-cells inhibitor-α, TNF-α = tumor necrosis factor-α, IL-6 = Interleukin-6.

### Protein Immunoassay

The preserved colon samples were taken out of the refrigerator. PIPA tissue lysate (Thermo Fisher Scientific Inc.) was used to extract the total protein from the tissue, and the protein concentration was determined using the microplate reader (Thermo Fisher Scientific Inc.) as instructed in the BCA kit (Beijing Solarbio Science and Technology Co. Ltd., Beijing, China). The protein samples were electrophoresed on SDS-PAGE (Thermo Fisher Scientific Inc.) and then transferred to polyvinylidene fluoride (PVDF) membranes (Thermo Fisher Scientific Inc.). The transferred membrane was sealed with 5% skim milk in TBST (Tris-buffered saline with Tween 20, Beijing Solarbio Science and Technology Co. Ltd.) for 1 h (25°C, 75 rpm, constant-temperature shaking incubator, Shidukai Instrument Equipment Co. Ltd., Shanghai, China) and then combined with the primary antibody for incubation overnight at 4°C. The antibody dilution ratios were as follows: β-actin (1:500), NF-κBp65 (1:1,000), IκB-α (1:500), TNF-α (1:500), p38 (1:500), p-p38 (1:1,000), JNK1/2 (1:500), and p-JNK1/2 (1:500). After the membranes were washed 3 times with a TBST buffer, a secondary antibody (Thermo Fisher Scientific Inc.) combining the blot with horseradish peroxidase was left for incubation for 1 h (37°C, 75 rpm). The chemiluminescent fluid (ECL Plus) kit (Beijing Solarbio Science and Technology Co. Ltd.) was used to detect immune complexes, and imaging was performed with the Tiangan chemiluminescence imaging system (Tanon Science and Technology Co. Ltd., Shanghai, China). The software Image J (National Institutes of Health, Bethesda, MD) was used to analyze the images, and β-actin was used as the internal reference protein to calculate the relative expression of the target protein (Zhou et al., 2019).

### Data Analysis

Graph Pad Prism 7.0 software (Graph Pad Software, La Jolla, CA) was used to calculate and analyze the results, which were expressed as mean standard deviations (SD). When *p* < 0.05, the result was considered statistically significant.

## Results

### Experimental Strains

**FIGURE 1:**
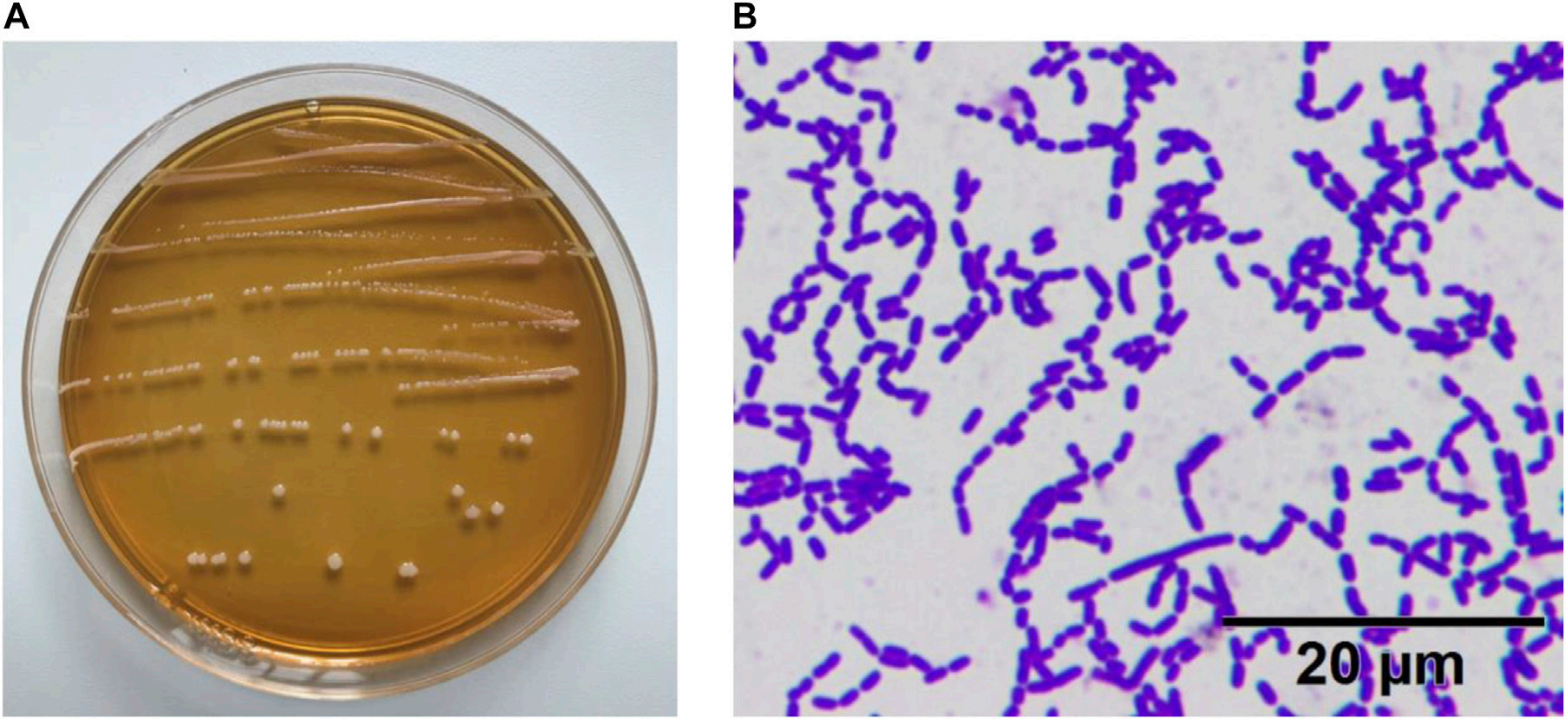
Morphological characteristics of experimental lactic acid bacterium ZS40. **(A)** colonies of the experimental strain. **(B)** Gram staining.

As shown in Figure 1, the colonies of the experimental strain on the plate were round, swollen, had neat edge, and the surface was moist and smooth. The result of gram staining was blue–purple staining, indicating that the experimental strain ZS40 was Gram-positive. No bud propagation was observed, indicating that ZS40 was a lactic acid bacteria.

### *In Vitro* Resistance Testing of *Lactobacillus fermentum* ZS40

The survival rate of ZS40 at artificial gastric juice pH 3.0 was 79.32%, and the growth efficiency when using 0.3% bile salt was 15.31%. The results showed that ZS40 exhibited adequate resistance *in vitro* and could be used in subsequent animal experiments.

### Colon Length

**FIGURE 2:**
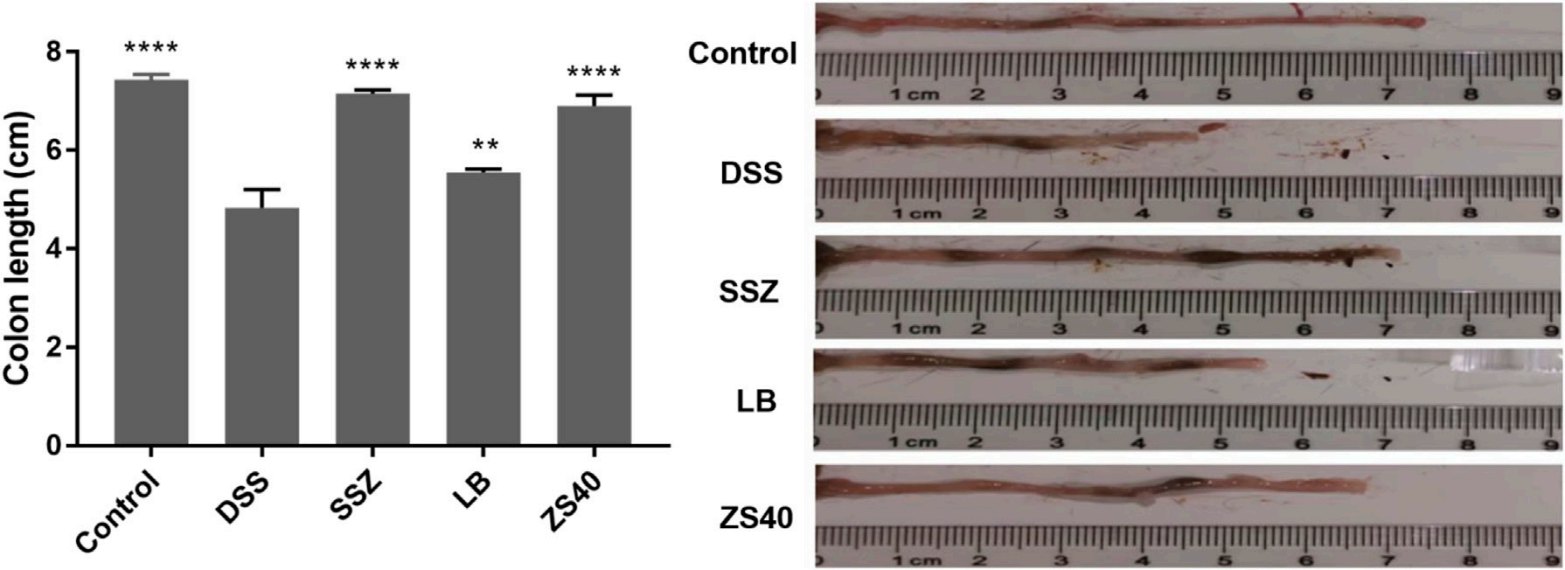
Colon length (cm) of experimental mice. Control group = gavage with saline, DSS group = 3% dextran sulfate sodium, SSZ group = 500 mg/kg body weight (b.w.) salicylazosulfapyridine treatment dose, LB group = 1.0 × 10^9^ CFU/kg body weight (b.w.) *L. bulgaricus* treatment dose, ZS40 group = 1.0 × 10^9^ CFU/kg body weight (b.w.) *L. fermentum* ZS40 treatment dose. Data are expressed as mean ± SD; **p < 0.01, ****p < 0.0001 compared with the DSS group.

The successful construction of mice colitis model was marked by shortening of the colon, edema of the intestinal mucosa, and visible ulcer formation. As shown in Figure 2, we measured the length of the colon to evaluate the effect of colitis on the colon length. The colon lengths of the mice in each group were as follows: 7.43 ± 0.11 cm in the control group, 4.83 ± 0.38 cm in the DSS group, 7.15 ± 0.08 cm in the SSZ group, 6.90 ± 0.22 cm in the ZS40 group, and 5.55 ± 0.07 cm in the LB group. These measurements were significantly longer than that in the DSS group. Compared with the DSS group, the colon length of the control group was significantly different (*p* < 0.0001). In addition, congestion and edema of the intestinal mucosa were also observed. The result showed that SSZ, ZS40, and LB could be used to treat the shortening of the mice colon and significantly alleviated congestion, edema, and ulcers.

### Histological Analysis

**FIGURE 3:**
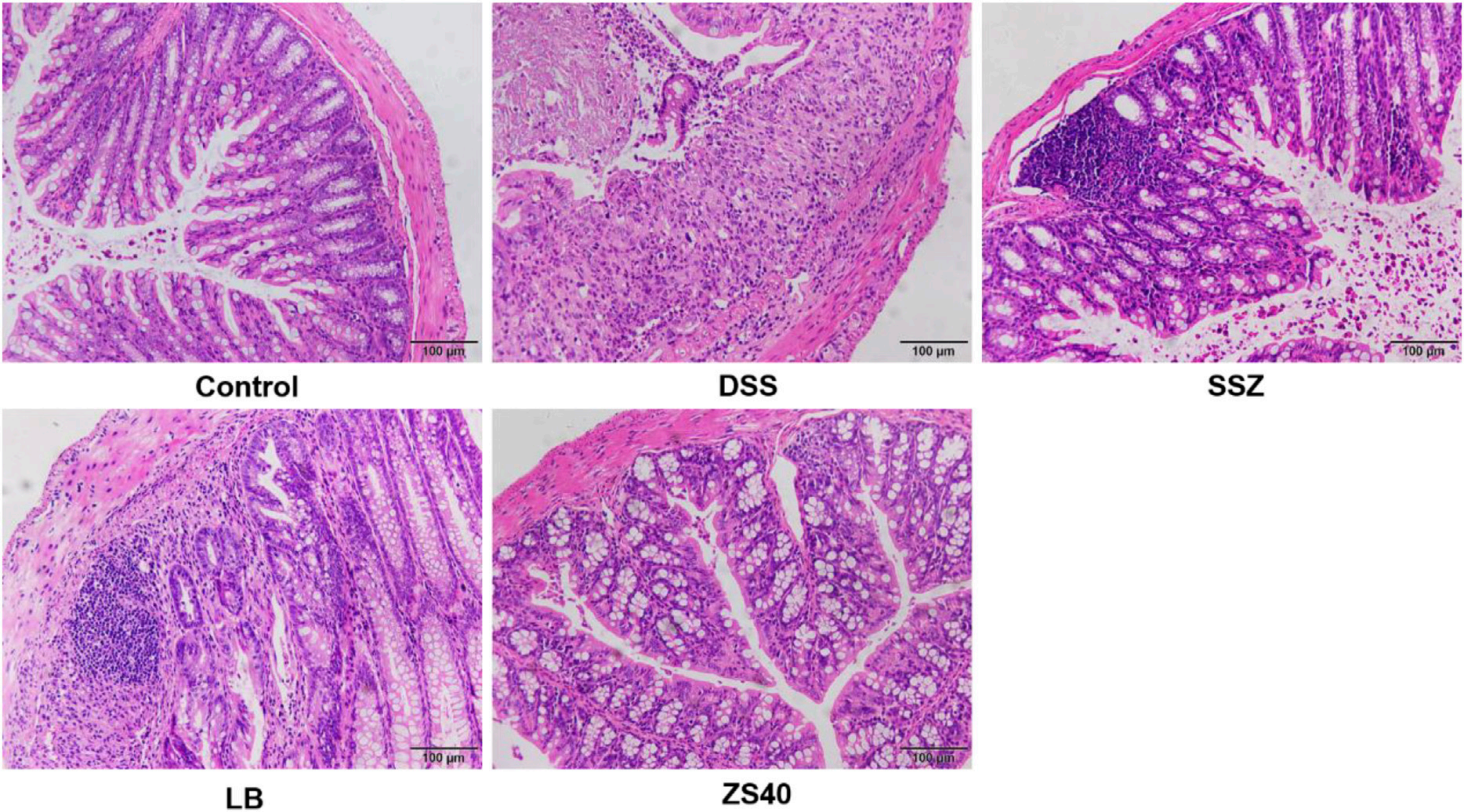
Histopathological observation of colon tissues. Magnification 200×.The length of the scale bar in each panel is 100 μm. Control group = gavage with saline, DSS group = 3% dextran sulfate sodium, SSZ group = 500 mg/kg body weight (b.w.) salicylazosulfapyridine treatment dose, LB group = 1.0 × 10^9^ CFU/kg body weight (b.w.) *Lactobacillus bulgaricus* treatment dose, ZS40 group = 1.0 × 10^9^ CFU/kg body weight (b.w.) *L. fermentum* ZS40 treatment dose.

Pathological tissues were observed after hematoxylin–eosin staining (Figure 3). In the control group, the colonic mucosal epithelial cells were intact, inflammatory cells were normal without infiltration, goblet cells were arranged neatly, and no hyperemia or edema was found. In the DSS group, epithelial cells were significantly damaged, the intestinal wall was thickened, edema, inflammatory cell infiltration, and goblet cells were reduced. After treatment with SSZ, ZS40 and LB, congestion, edema, cell infiltration and erosion were relieved. ZS40 improved the colon tissues the most. ZS40 also improved the DSS-induced colon injury and prevented colitis.

### Analysis of Serum Biochemical Indicators in Mice

**FIGURE 4:**
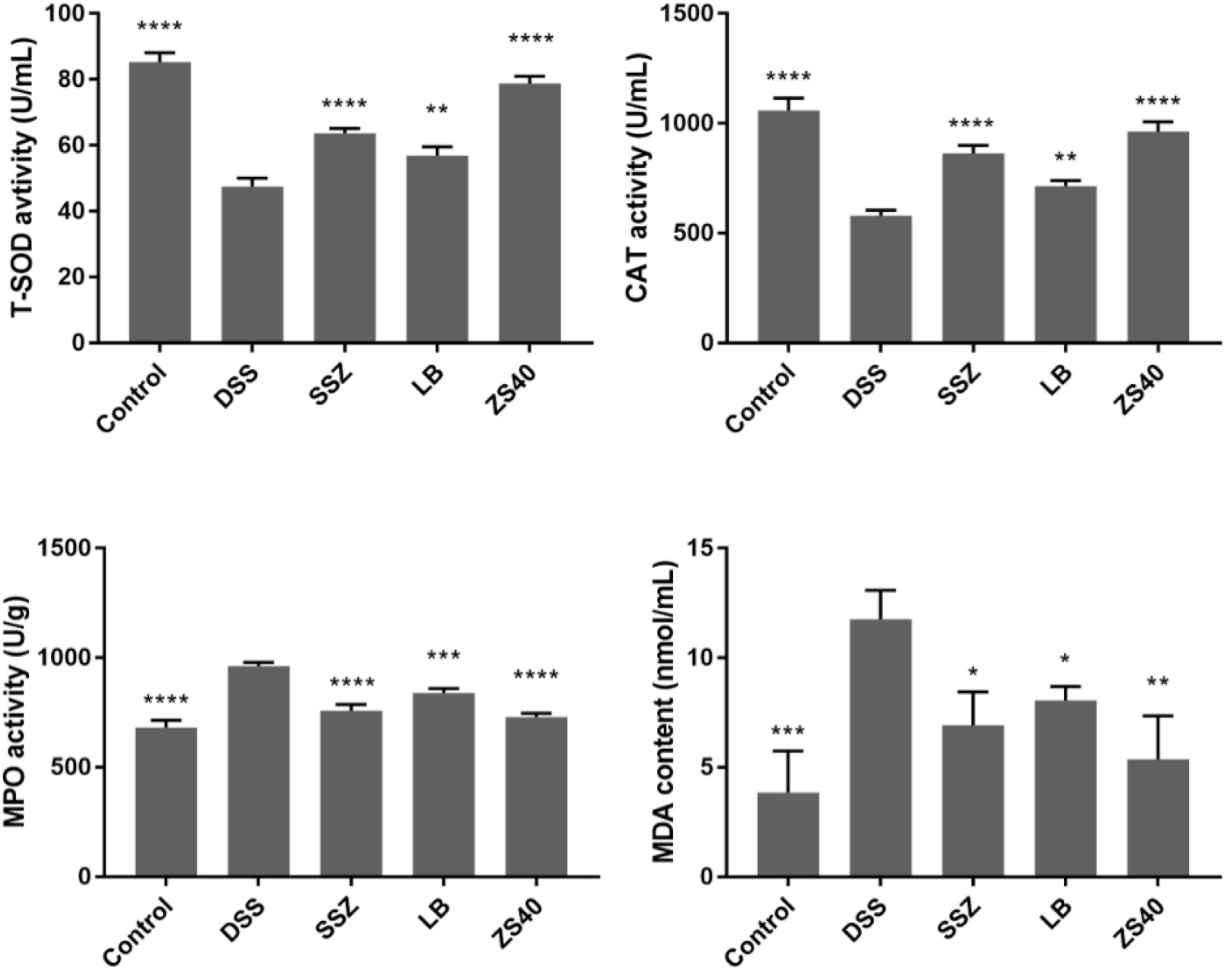
The levels of superoxide dismutase (T-SOD), myeloperoxidase (MPO), catalase (CAT) and malondialdehyde (MDA) in serum of mice. Control group = gavage with saline, DSS group = 3% dextran sulfate sodium, SSZ group = 500 mg/kg body weight (b.w.) salicylazosulfapyridine treatment dose, LB group = 1.0 × 10^9^ CFU/kg body weight (b.w.) *L. bulgaricus* treatment dose, ZS40 group = 1.0 × 10^9^ CFU/kg body weight (b.w.) *L. fermentum* ZS40 treatment dose. Data are expressed as mean ± SD; *p < 0.05, **p < 0.01, ***p < 0.001, ****p < 0.0001 compared with the DSS group.

As shown in Figure 4, the DSS group exhibited the lowest serum T-SOD and CAT activity levels but the highest MPO and MDA contents, whereas the control group showed the opposite trend. In this study, SSZ, ZS40, and LB could increase the activity of T-SOD and CAT, and reduce the activity of MPO and the content of MDA. These results indicated that they could scavenge hydroxyl radicals, thereby alleviating colitis in mice. The ZS40 showed the closest T-SOD, CAT, MPO, and MDA activity levels to those in the normal group, indicating that ZS40 exerted the best effect on the mice.

### Determination of IL-1β, IL-6, IL-10, and TNF-α Levels in Serum

**FIGURE 5:**
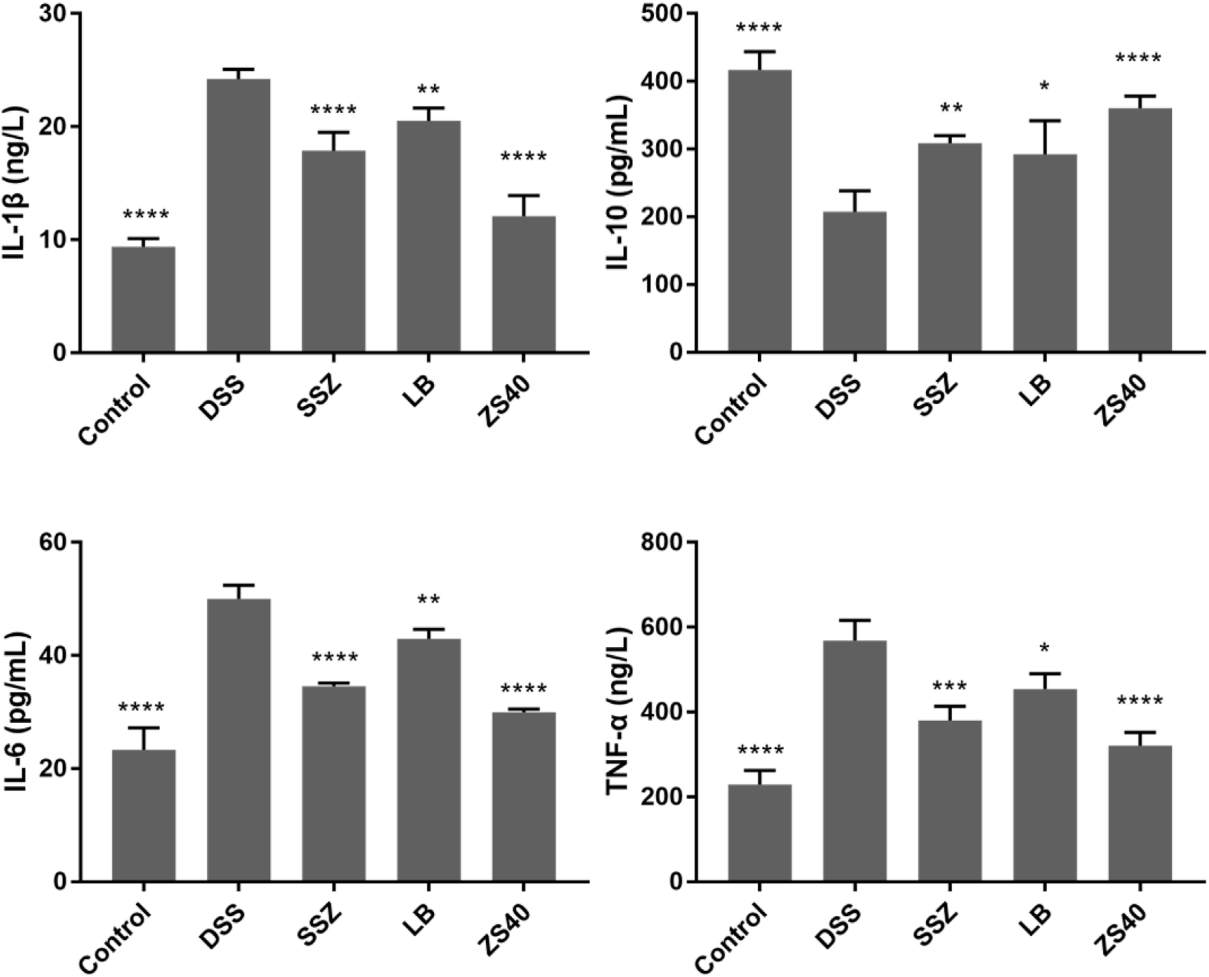
Cytokine levels (IL-1β, IL-6, IL-10, and TNF-α) in serum of mice. Control group = gavage with saline, DSS group = 3% dextran sulfate sodium, SSZ group = 500 mg/kg body weight (b.w.) salicylazosulfapyridine treatment dose, LB group = 1.0 × 10^9^ CFU/kg body weight (b.w.) *L. bulgaricus* treatment dose, ZS40 group = 1.0 × 10^9^ CFU/kg body weight (b.w.) *L. fermentum* ZS40 treatment dose. Data are expressed as mean ± SD; *p < 0.05, **p < 0.01, ***p < 0.001, ****p < 0.0001 compared with the DSS group.

The levels of the pro-inflammatory cytokines IL-1β, IL-6, and TNF-α increased significantly in the DSS group, and the opposite trend was observed in the control group (Figure 5). Treatment with ZS40 could inhibit the levels of IL-1β, IL-6, IL-12, and TNF-α, and promote IL-10. The serum cytokine levels of the mice were closer to that of the normal group. This result showed that ZS40 could inhibit the pro-inflammatory factors and promote the anti-inflammatory cytokines, thereby reducing inflammation.

### Effect of *Lactobacillus fermentum* ZS40 on the NF-κB Pathway

**FIGURE 6:**
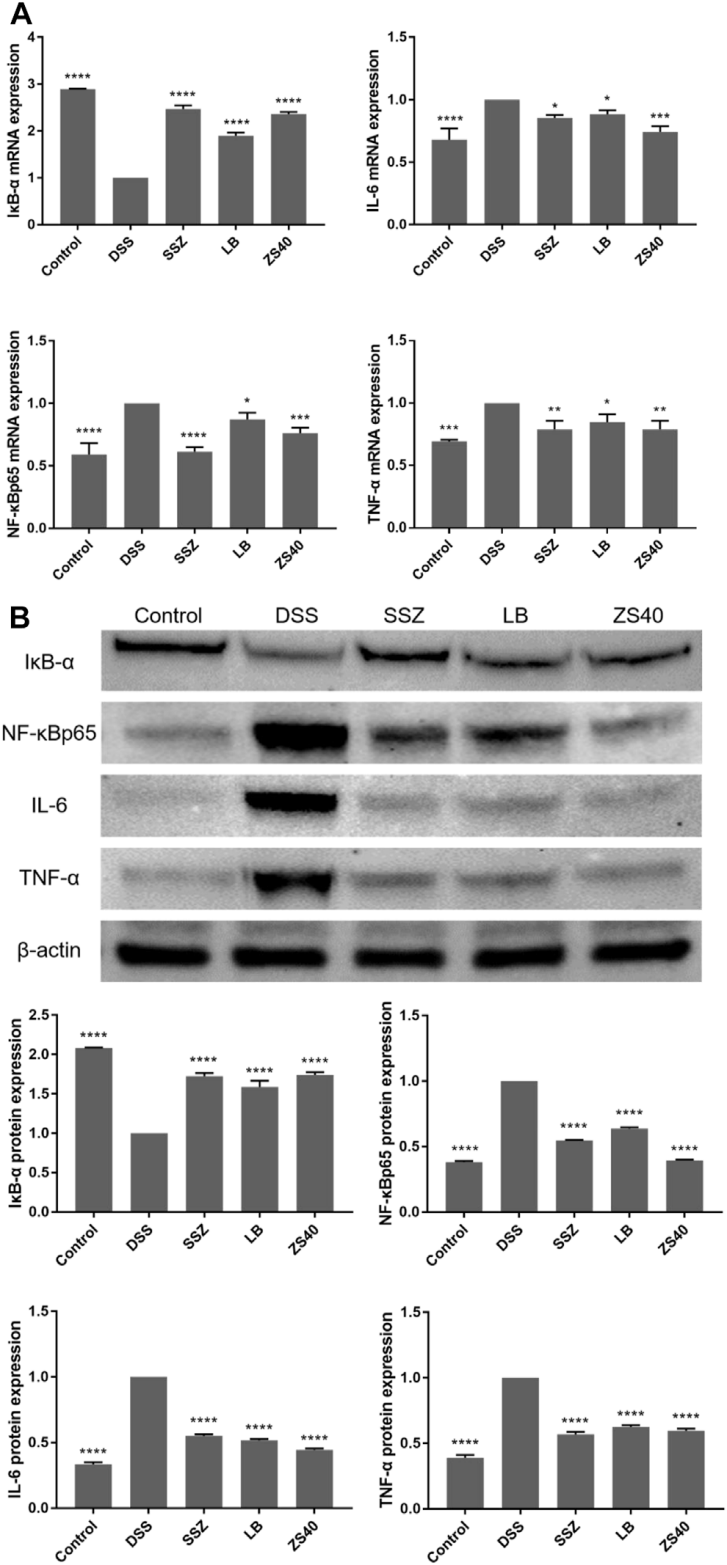
The mRNA and protein expression levels of IκB-α, NF-κB, IL-6 and NF-κB inhibitor-α (IκB-α) in colon of mice. **(A)** mRNA expression, **(B)** protein expression. Control group = gavage with saline, DSS group = 3% dextran sulfate sodium, SSZ group = 500 mg/kg body weight (b.w.) salicylazosulfapyridine treatment dose, LB group = 1.0 × 10^9^ CFU/kg body weight (b.w.) *L. bulgaricus* treatment dose, ZS40 group = 1.0 × 10^9^ CFU/kg body weight (b.w.) *L. fermentum* ZS40 treatment dose. Data are expressed as mean ± SD; *p < 0.05, **p < 0.01, ***p < 0.001, ****p < 0.0001 compared with the DSS group.

To assess whether the inhibitory effect of ZS40 on colitis was mediated by the NF-κB pathway, we measured the relative expression of IκB-α, NF-κBp65, IL-6, and TNF-α mRNA and protein in colon tissues (Figure 6). Compared with the control group, the relative expression of NF-κBp65, IL-6, TNF-α mRNA and protein in the colon tissue of the DSS group increased, and the relative expression of IκB-α mRNA and protein decreased. The ZS40 group was the opposite, which was closer to the control group.

### Effect of *Lactobacillus fermentum* ZS40 on the MAPK Pathway

**FIGURE 7:**
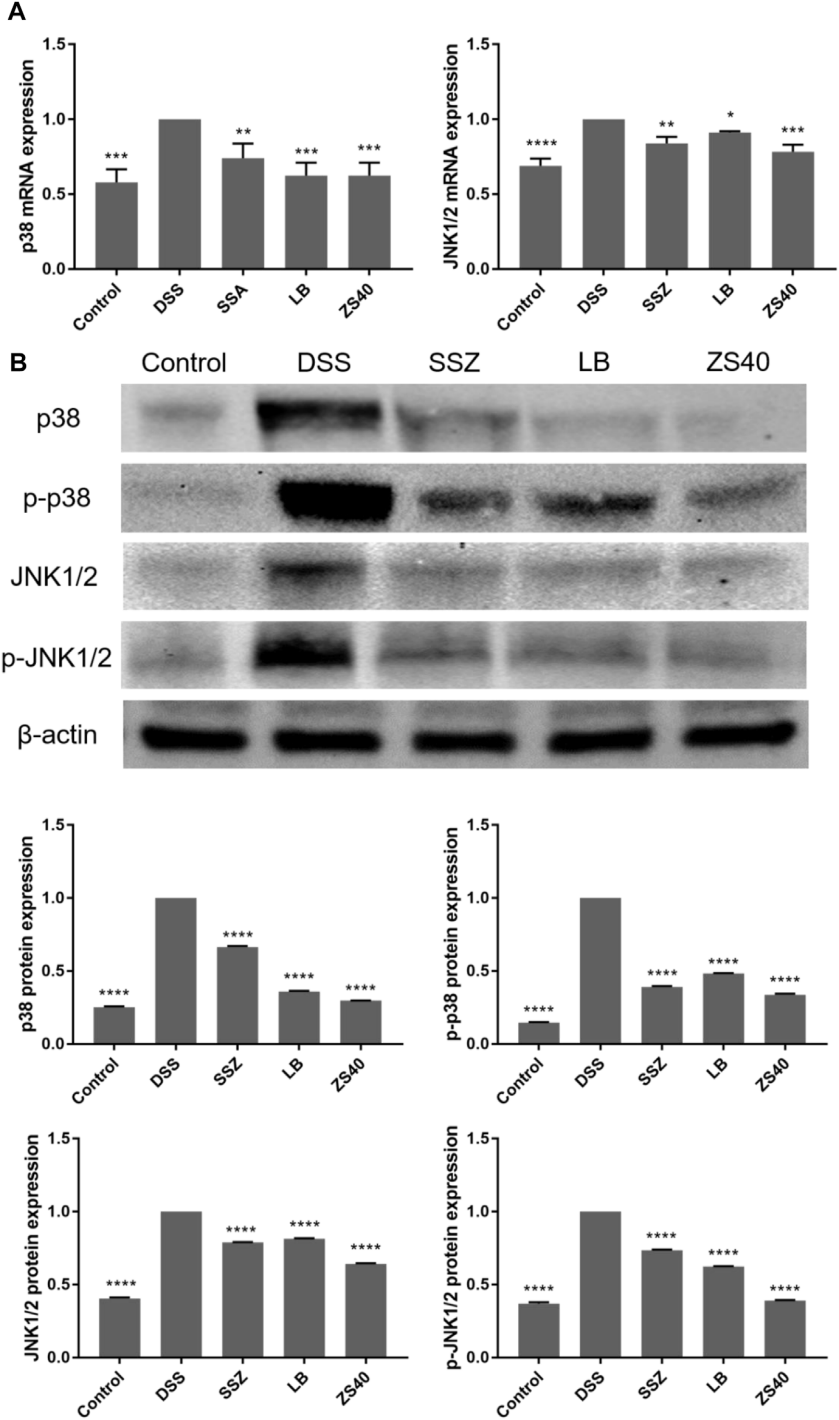
The mRNA expression levels of p38 and JNK1/2 in colon of mice **(A)**. The protein expression levels of p38, p-p38, JNK1/2 and p-JNK1/2 in colon of mice **(B)**. Control group = gavage with saline, DSS group = 3% dextran sulfate sodium, SSZ group = 500 mg/kg body weight (b.w.) salicylazosulfapyridine treatment dose, LB group = 1.0 × 10^9^ CFU/kg body weight (b.w.) *L. bulgaricus* treatment dose, ZS40 group = 1.0 × 10^9^ CFU/kg body weight (b.w.) *L. fermentum* ZS40 treatment dose. Data are expressed as mean ± SD; *p < 0.05, **p < 0.01, ***p < 0.001, ****p < 0.0001 compared with the DSS group.

To assess whether the inhibitory effect of ZS40 on colitis was mediated by the MAPK pathway, we determined the relative expression of p38 and JNK1/2 mRNA, as well as the relative protein expression of p38, p-p38, JNK1/2, and p-JNK1/2 in colon tissues (Figure 7). The relative mRNA expression of p38 and JNK1/2 and the relative protein expression of p38, p-p38, JNK1/2, and p-JNK1/2 in colon tissues in the DSS group increased relative to those in the control group. ZS40 group were more similar to those of the control group.

## Discussion

Ulcerative colitis is a chronic non-specific inflammation with complicated etiology and pathogenesis. Considerable data have shown that probiotics have different degrees of therapeutic relief and alleviating effects on colitis. Zhou et al. proved that *L. fermentum* CQPC04 exerted a protective effect on DSS-induced colitis in mice and was related to the nuclear factor-κB signaling pathway (Zhou et al., 2019). Michael et al. demonstrated that *L. plantarum* 299 V could be used to treat and prevent spontaneous colitis in mice with IL-10 deficiency (Schultz et al., 2002). In addition, probiotics are normal inhabitants of the human body, exhibiting high safety and low toxicity, and involving no side effects (Jung et al., 2017). Probiotics can potentially be used as a treatment in the future.

In the present study, 3% DSS was used to construct a mouse model of colitis. The modeling principle of 3% DSS was to destroy the intestinal mucosa, change the permeability of the intestinal mucosa, and allowed macromolecules to enter the intestinal mucosa, causing tissue damage. In addition, the modeling method was convenient and simple to operate. It exhibited good reproducibility, which was highly similar to the prevalence of human colitis (Chassaing et al., 2014). During the modeling process, the body weight of the colitis model mice showed a downward trend. After anatomy, it was found that the colon was significantly shortened, with edema, bleeding, and small ulcer formation. HE staining showed irregular morphological changes, inflammatory cell infiltration, and decrease of goblet cells in the damaged colon tissue, indicating that the established mice colitis model was successful. In this study, ZS40 could relieve colon shortening and inflammatory lesions of colon tissues. Its effect was similar to sulfasalazine, which is commonly used to treat colitis.

The effect of ZS40 on oxidative stress in the serum of colitis was evaluated by monitoring antioxidant indicators and lipid peroxidation biomarkers, such as T-SOD, CAT, MPO, and MDA. T-SOD exists in the antioxidant enzyme system, which stabilizes cell membranes by scavenging free radicals and inhibiting peroxidation (Mrowicki et al., 2016). T-SOD activity is an important indicator of the anti-inflammatory response of the body. CAT is an oxygen active substance scavenger, its activity clearly reflects the body’s ability to scavenge hydroxyl free radicals (Tomusiak-Plebanek et al., 2018). MPO is an enzyme in neutrophils whose activity indicates the severity of neutrophil infiltration and colitis (Qi-Yi et al., 2012). MDA is a type of lipid peroxide, which is formed when free radicals attack biological membranes. It reflects the degree of lipid peroxidation (Macotpet et al., 2013). In this study, the serum T-SOD and CAT activities in the DSS group decreased, and the MDA content and MPO activity increased. The serum T-SOD and CAT activity levels in the SSZ, ZS40, and LB groups increased, while MPO activity and MDA content decreased. In addition, the enhancing effect of ZS40 was superior. The results showed that ZS40 could increase the activity of antioxidant enzymes, improve the ability of the body to scavenge hydrogen and oxygen-free radicals, and ultimately reduce the oxidative damage to the mice colon.

Cytokines are a class of small molecular proteins that transmit signals. They mainly regulate immune response, mediate inflammation, and participate in tissue repair (Bamias et al., 2014). Two types of cytokines are involved in inflammatory response: anti-inflammatory cytokines, including IL-10, and pro-inflammatory cytokines, including IL-1β, IL-6, and TNF-α (Tao et al., 2009). Neutrophil infiltration of colonic mucosa and macrophages secrete a large number of pro-inflammatory cytokines, and inhibit the secretion of anti-inflammatory cytokines during inflammation, thereby exacerbating the inflammatory state (Shukla et al., 2016). IL-1β is generally produced by monocytes and macrophages, but it can also be synthesized and secreted by neutrophils, plasma cells, and other nucleated cells after being stimulated by external antigens (Mccarty and Frishman, 2014). IL-1β promotes the activation and aggregation of inflammatory cells, increases the permeability of epithelial and endothelial cells, intensifies the inflammation of intestinal mucosa, and mediates hyperalgesia in inflammation (Park et al., 2019). IL-6 is a pro-inflammatory factor produced by activated T cells and fibroblasts that activates the NF-κB signaling pathway and promote the expression of intercellular adhesion molecules (Oshima and Oshima, 2012; Guo et al., 2016). Overexpression of IL-6 can affect intestinal epithelial cells, change their permeability, infiltrate neutrophils on the mucosa, and induce or aggravate intestinal inflammation (Al-Sadi et al., 2014). TNF-α, which is produced by monocytes and macrophages, appears earliest during inflammation. The effect on colitis is to increase the permeability of vascular endothelial cells, and accumulate inflammatory cells at the inflammation site, causing inflammatory cell infiltration and tissue edema (Zhou et al., 2006; Chae et al., 2019). TNF-α combined with the pro-inflammatory cytokine IFN-γ can structurally change intestinal epithelial cells. IL-10 is secreted by macrophages, dendritic cells, and T cells, and plays an important role in maintaining the stability of the intestinal environment. The main physiological function of IL-10 is to inhibit neutrophils, reduce the expression of pro-inflammatory factors, and protect tissues and organs from injury (Keubler et al., 2015). In this study, ZS40 could significantly inhibit the production of pro-inflammatory cytokines such as IL-1β, IL-6, TNF-α, and promote the production of anti-inflammatory cytokine IL-10.

NF-κB and MAPK are two important pathways involved in the regulation of inflammation. NF-κB plays an important role in the regulation of many immune and inflammatory responses (Yoo et al., 2017). It normally binds to its inhibitory protein IκB and exists as an inactive dimer in the cytoplasm. However, activation of NF-κB induces inhibitory protein kinase and dephosphorylation of IκB in response to inflammatory stimuli (Ju et al., 2015). After the separation of NF-κB and IκB, NF-κB is activated and transferred to the nucleus to promote the induction and secretion of numerous pro-inflammatory cytokines (such as TNF-α and IL-6), among which the transcription TNF-α and IL-6 has been proven to be affected by the NF-κB pathway (Ren et al., 2018). In addition, overexpression of TNF-α can activate NF-κB and exacerbate the inflammatory process. MAPK is a class of intracellular serine/threonine protein kinases, which includes three subtypes: ERK, p38, and JNK (Song et al., 2015). The p38 mediates inflammation, cell apoptosis, etc. After activation, p38 can transfer signals from the cytoplasm to the nucleus, promoting cell metabolism and cell survival. JNK is a type of mitogen, also known as stress-activated protein kinase or c-Jun N-terminal kinase. It plays an important role in apoptosis and can be activated by various stimuli (Heldin and Moustakas, 2016). In addition, JNK1/2 is an important member of MAPK. JNK1/2 and p-p38 are the main factors that regulate the expression of inflammatory cytokines (Wang et al., 2015). Activated by growth factors, cytokines, inflammation and stress, MAPK signaling pathway is involved in the regulation of cell proliferation, differentiation, metastasis, apoptosis, cell cycle and inflammation (Zhang et al., 2016). Studies have shown that NF-κB is one of the downstream components in the MAPK signal transduction pathway (Gao et al., 2020). Therefore, the activation of the MAPK signaling pathway can also indirectly activate the NF-κB signaling pathway and induce the production of inflammatory factors. In summary, blocking the activation of NF-κB and MAPK signaling pathways to down-regulate the expression of inflammation-related genes and proteins is an ideal way to treat colitis. We evaluated the effect of ZS40 on the activation of NF-κB and MAPK. ZS40 could down-regulate the relative expression of NF-κBp65, IL-6, and TNF-α mRNA and protein, up-regulate the relative expression of IκB-α mRNA and protein. It could also down-regulate the expression of p38 and JNK1/2 mRNA, and the expression of p38, p-p38, JNK1/2, and p-JNK1/2 protein. Inflammation is alleviated by inhibiting the activation of NF-κB and MAPK pathways.

## Conclusion

ZS40 is a new type of *Lactobacillus* isolated from traditionally fermented yak yogurt in Paleksu Kaisk grassland in Zhaosu County, Xinjiang, China. The *in vitro* resistance test showed that ZS40 had good *in vitro* resistance. The strain exhibited good *in vitro* resistance, as determined by testing. ZS40 could inhibit 3% DSS-induced mice colon shortening, colon damage, and intestinal wall thickening. It improved the activity of T-SOD and CAT, reduced MPO and MDA, regulated the balance of pro-inflammatory cytokines and anti-inflammatory cytokines, inhibited the activation of NF-κB and MAPK signaling pathways, and ultimately relieved inflammation. In summary, the strain can effectively alleviate the symptoms of colitis in mice and thus can be used to prevent colitis.

## Data Availability

The original contributions presented in the study are included in the article/Supplementary Material, further inquiries can be directed to the corresponding authors.
