# Supplementary figures and images for: Lactobacillus fermentum ZS40 Ameliorates Inflammation in Mice With Ulcerative Colitis Induced by Dextran Sulfate Sodium
Source: Front Pharmacol. 2021 Nov 19;12:700217. doi: 10.3389/fphar.2021.700217 (PMC8640127; doi:10.3389/fphar.2021.700217)

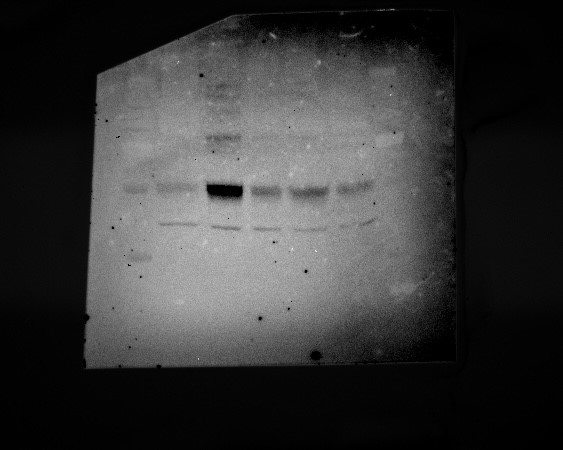

Supplement: Supplementary file 1 [file DataSheet1.ZIP › western blot/IL-6.jpg]

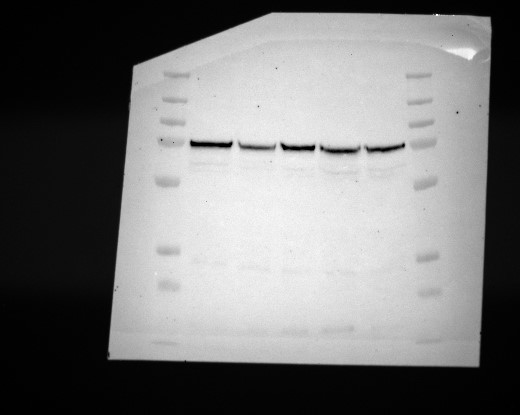

Supplement: Supplementary file 1 [file DataSheet1.ZIP › western blot/IκB-α.jpg]

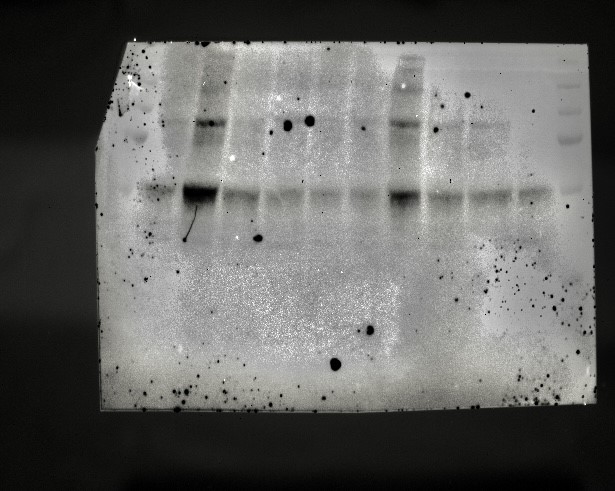

Supplement: Supplementary file 1 [file DataSheet1.ZIP › western blot/JNK1 2.jpg]

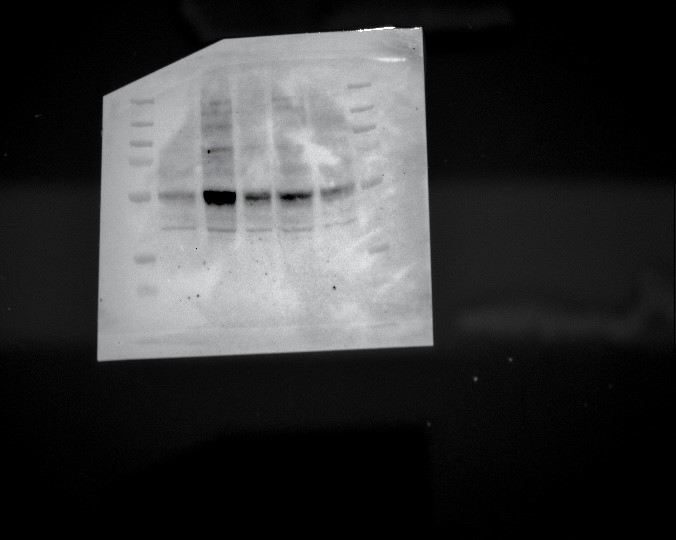

Supplement: Supplementary file 1 [file DataSheet1.ZIP › western blot/NF-κB.jpg]

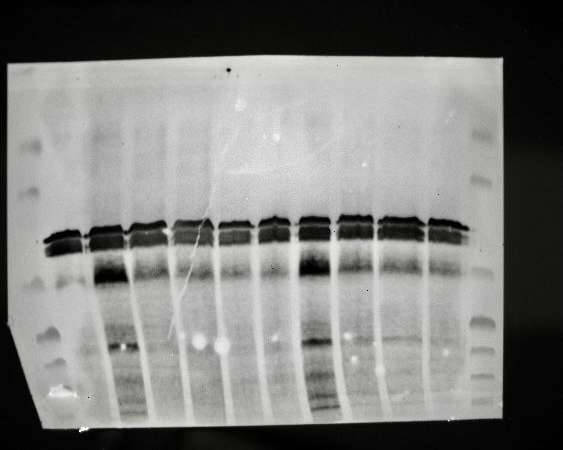

Supplement: Supplementary file 1 [file DataSheet1.ZIP › western blot/p-JNK1 2.jpg]

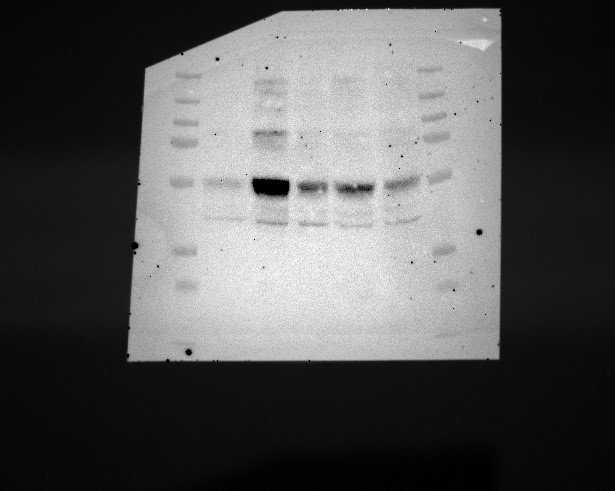

Supplement: Supplementary file 1 [file DataSheet1.ZIP › western blot/p-p38.jpg]

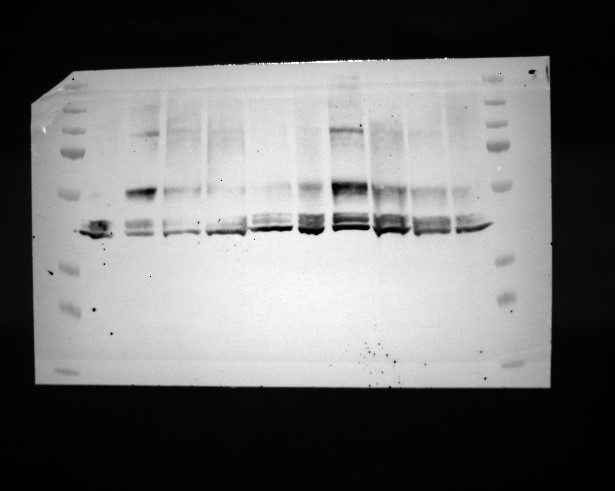

Supplement: Supplementary file 1 [file DataSheet1.ZIP › western blot/p38.jpg]

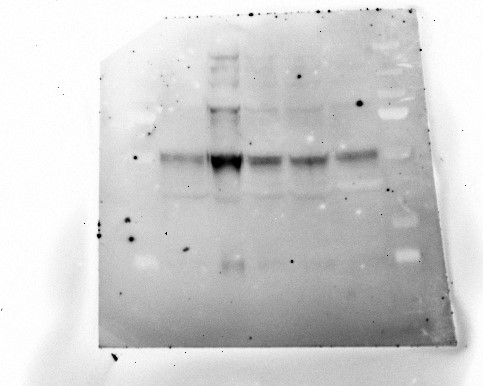

Supplement: Supplementary file 1 [file DataSheet1.ZIP › western blot/TNF-α.jpg]

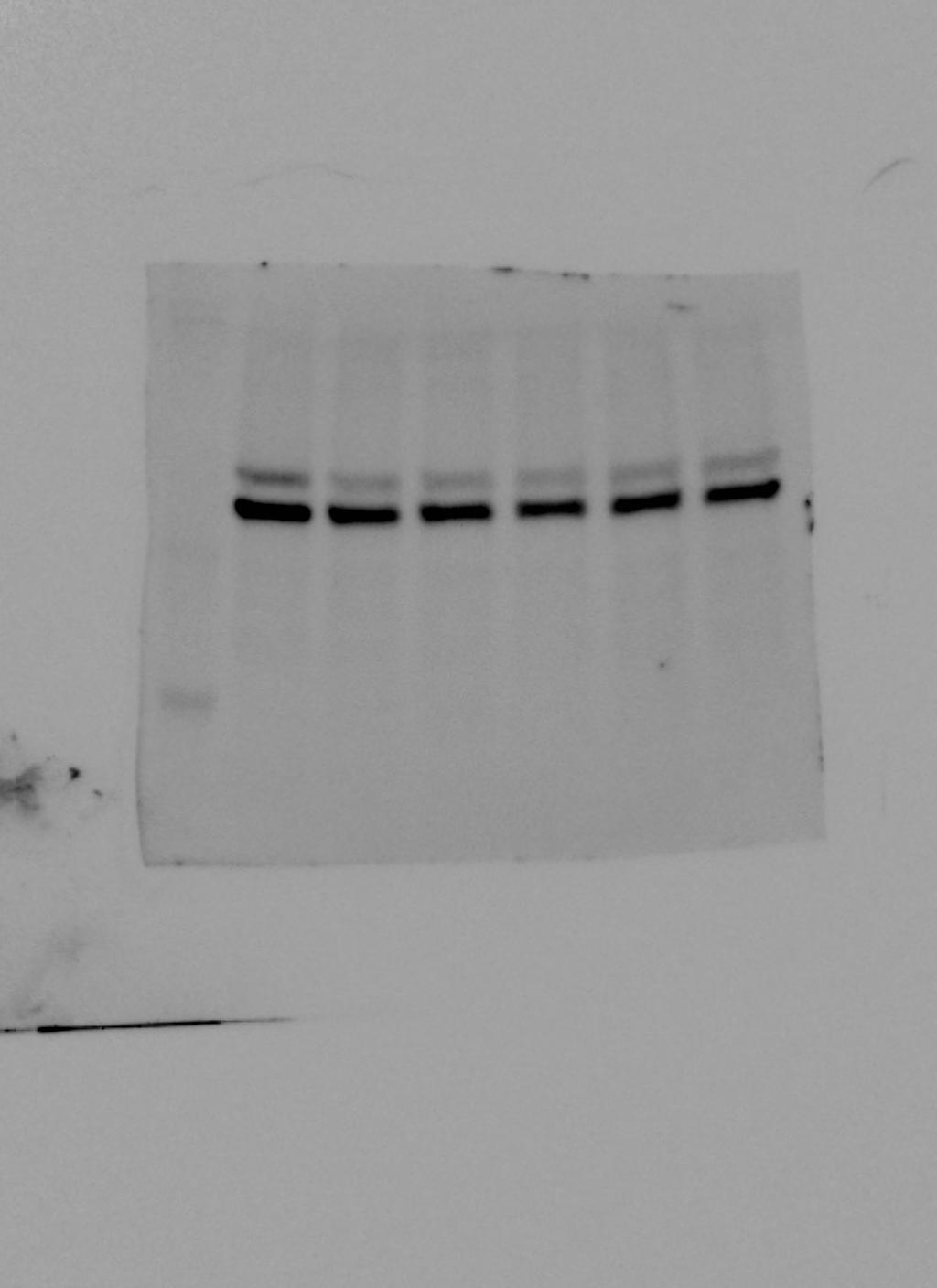

Supplement: Supplementary file 1 [file DataSheet1.ZIP › western blot/β-actin.JPG]

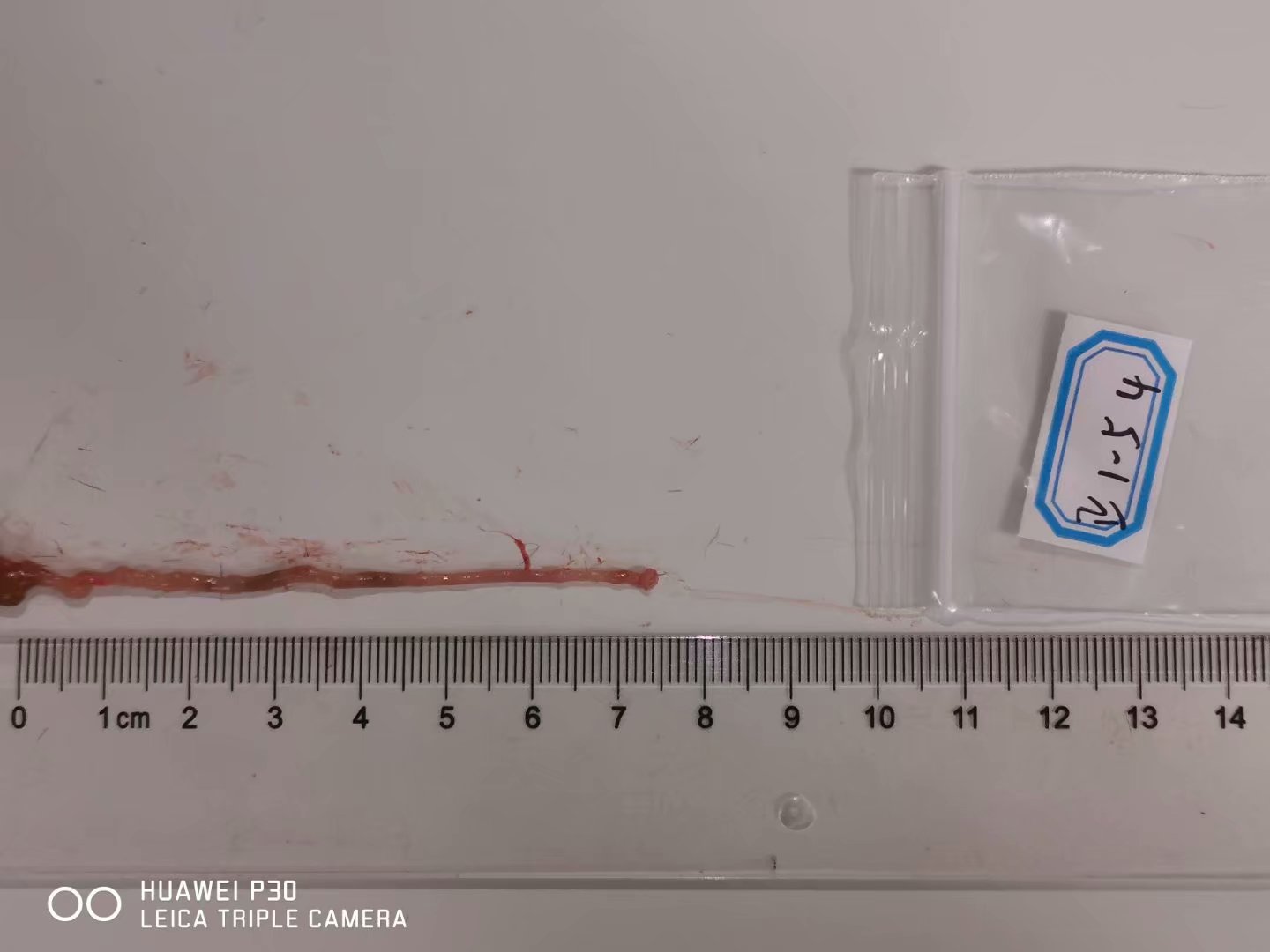

Supplement: Supplementary file 1 [file DataSheet1.ZIP › colon length/Control.jpg]

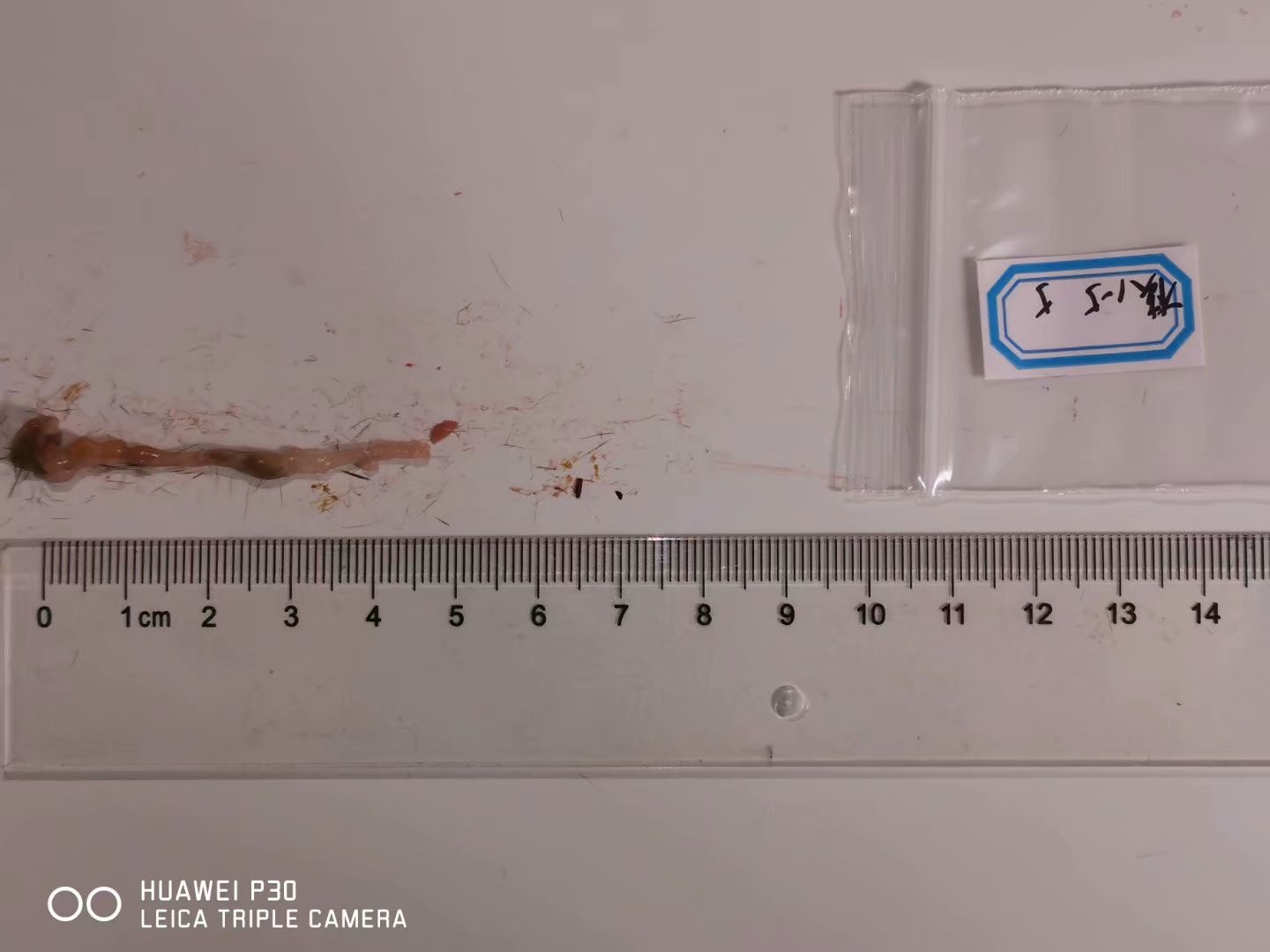

Supplement: Supplementary file 1 [file DataSheet1.ZIP › colon length/DSS.jpg]

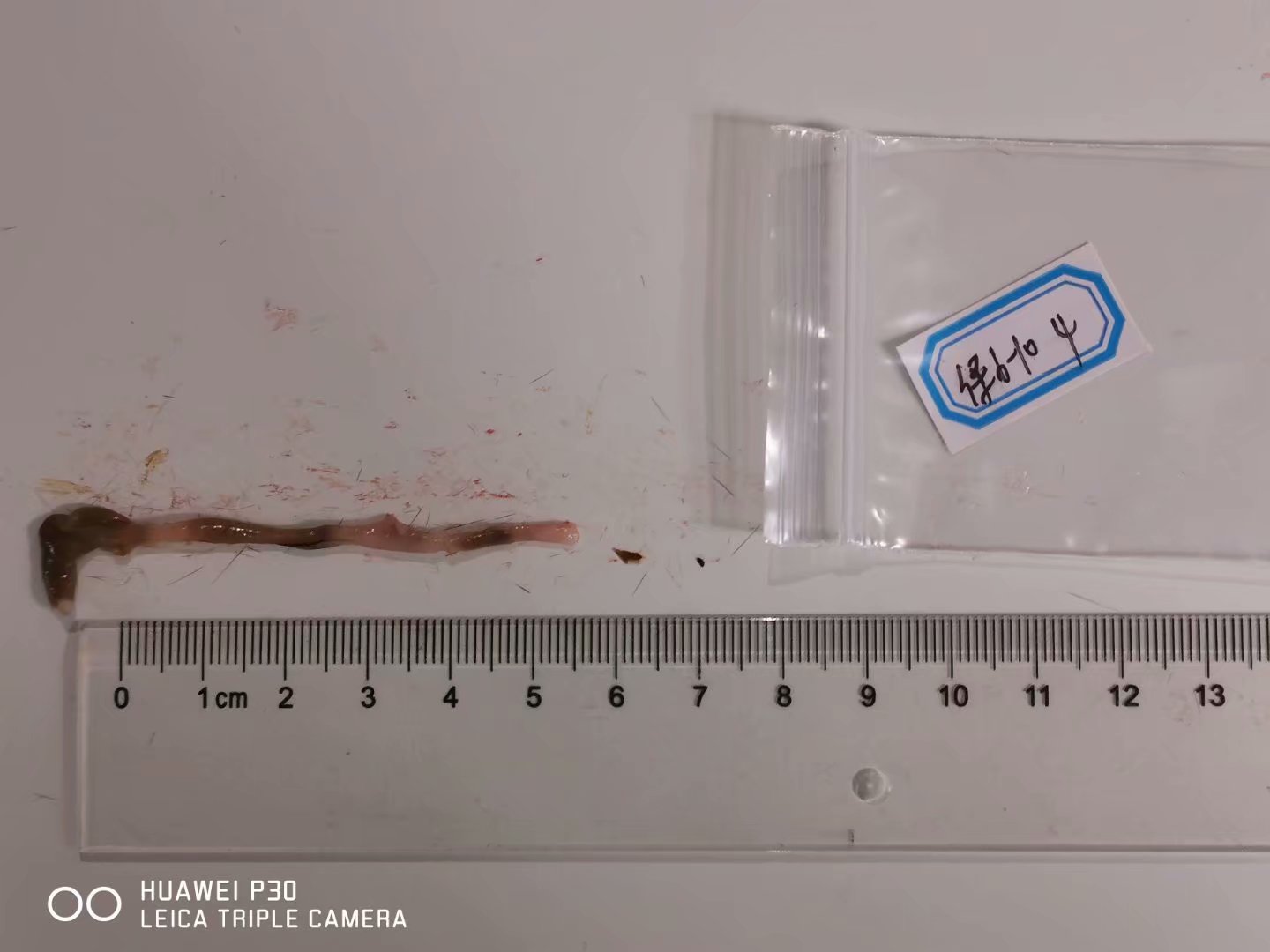

Supplement: Supplementary file 1 [file DataSheet1.ZIP › colon length/LB.jpg]

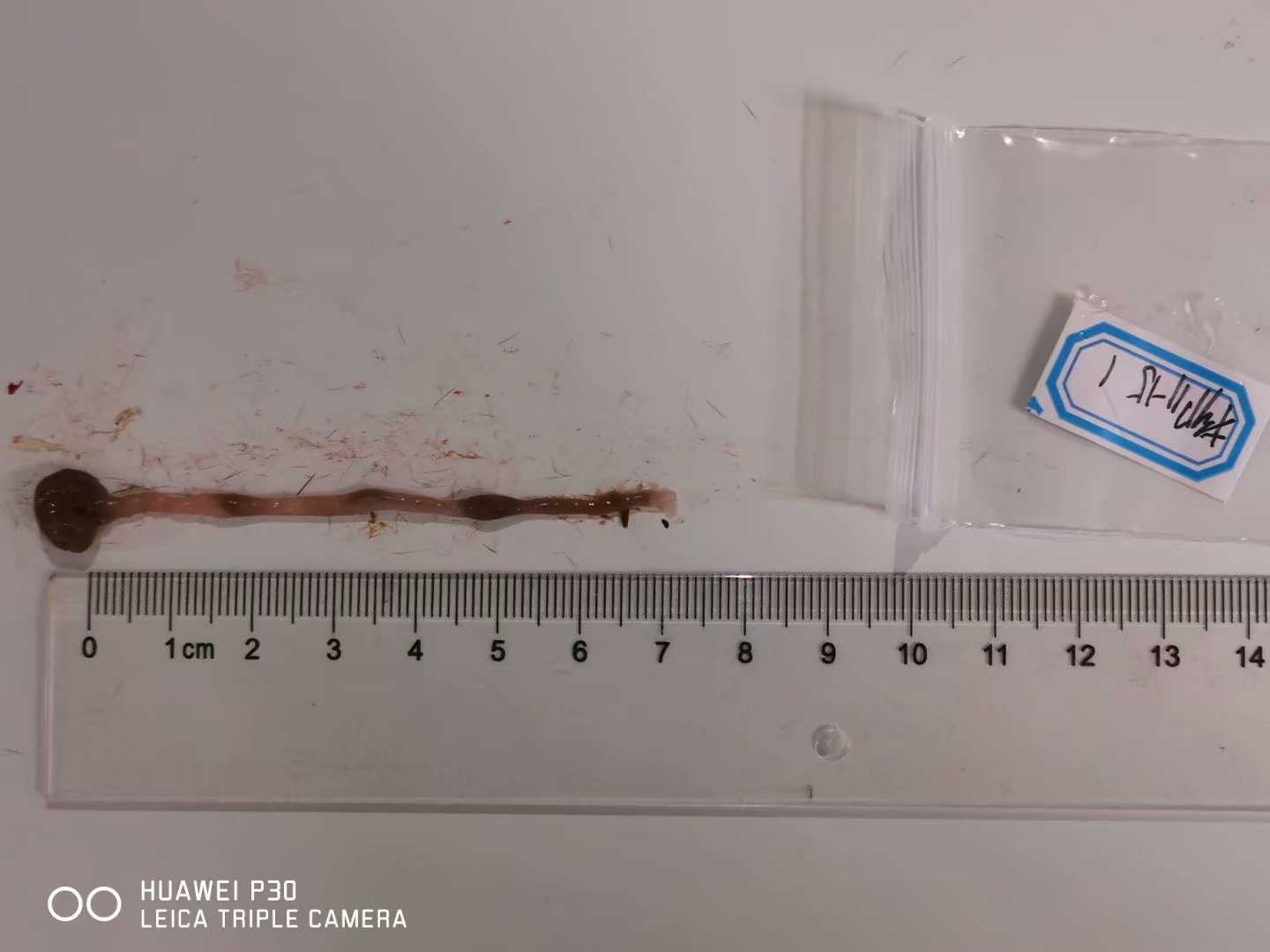

Supplement: Supplementary file 1 [file DataSheet1.ZIP › colon length/SSZ.jpg]

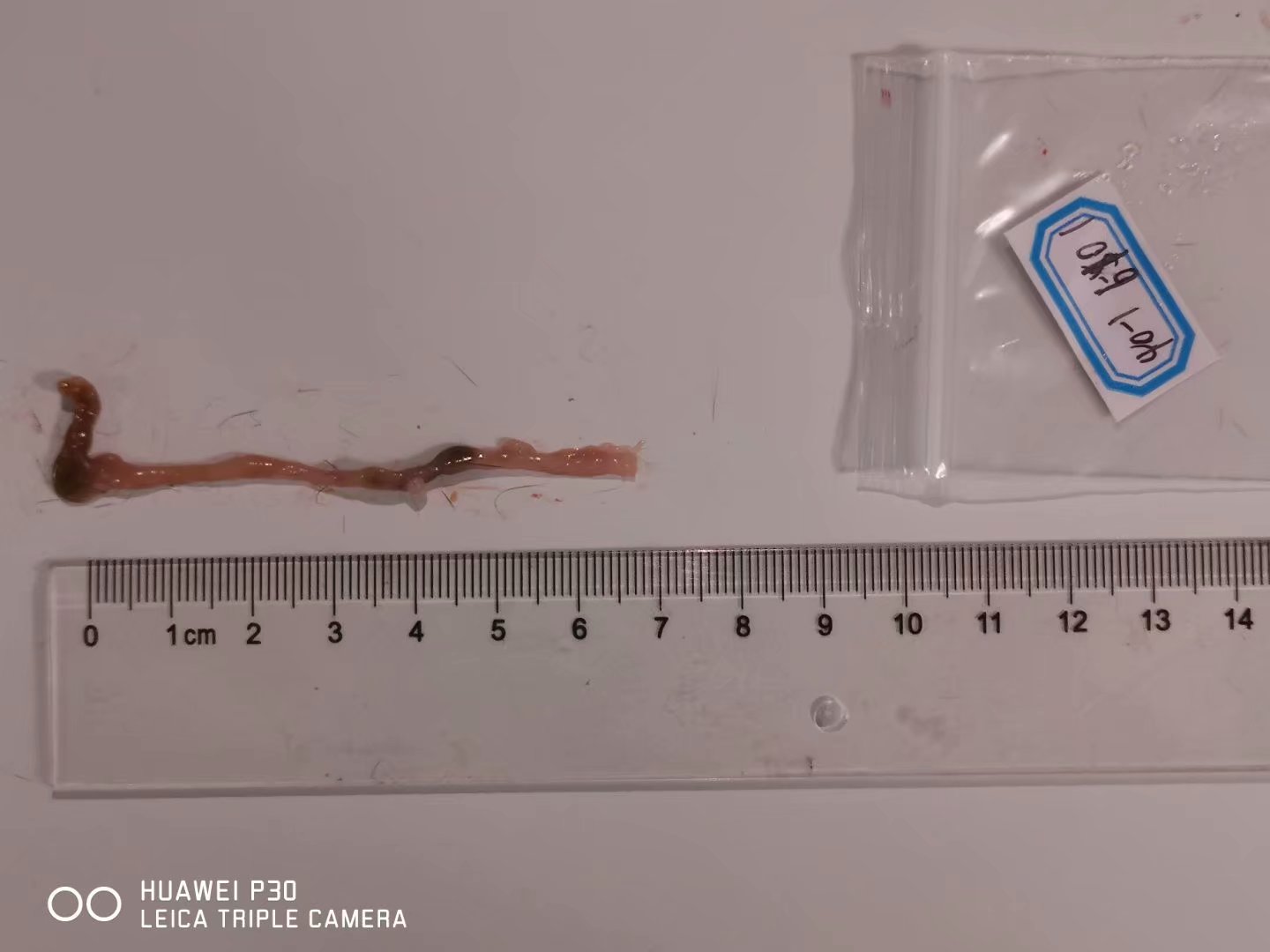

Supplement: Supplementary file 1 [file DataSheet1.ZIP › colon length/ZS40.jpg]

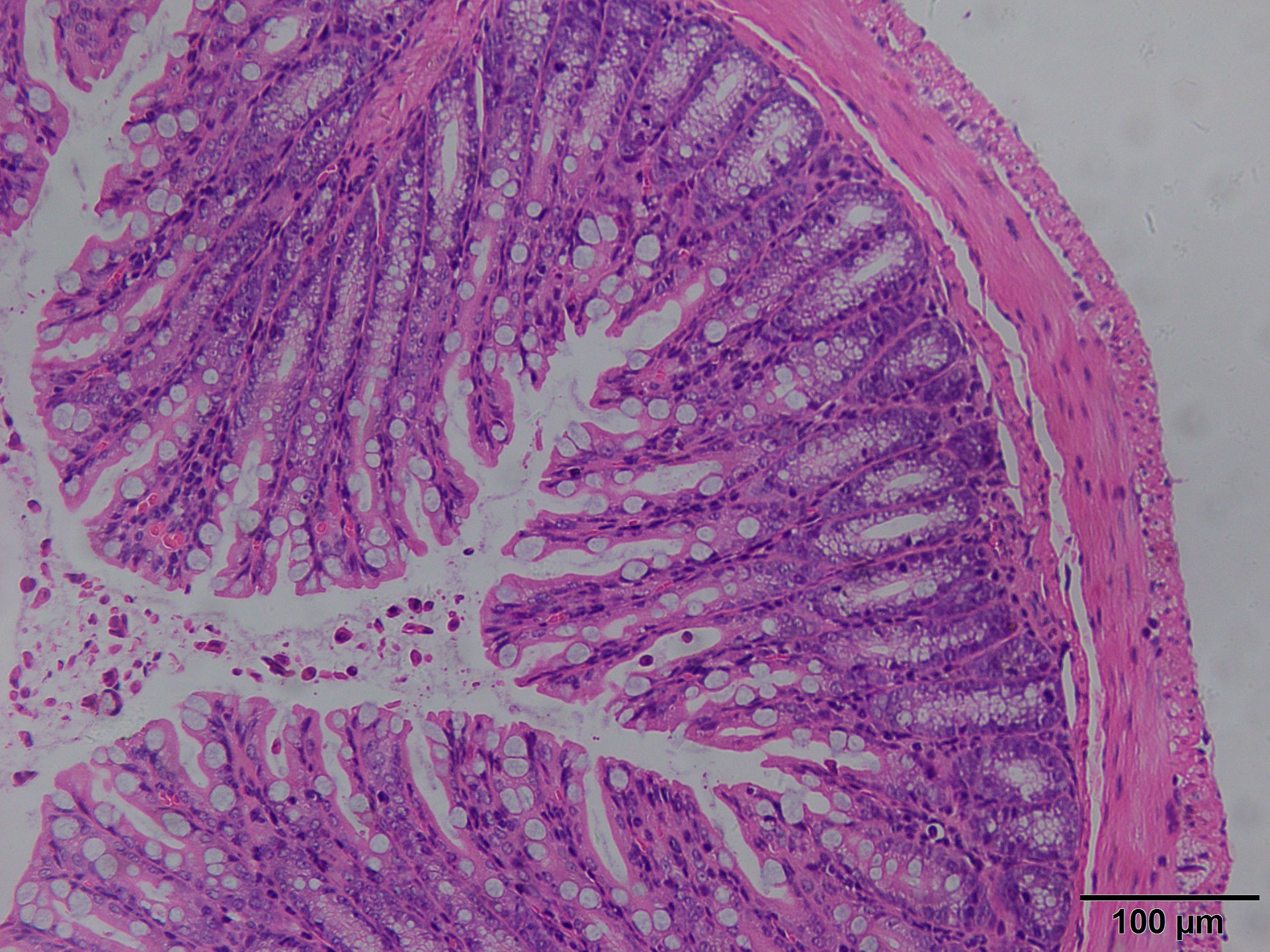

Supplement: Supplementary file 1 [file DataSheet1.ZIP › HE/Control.jpg]

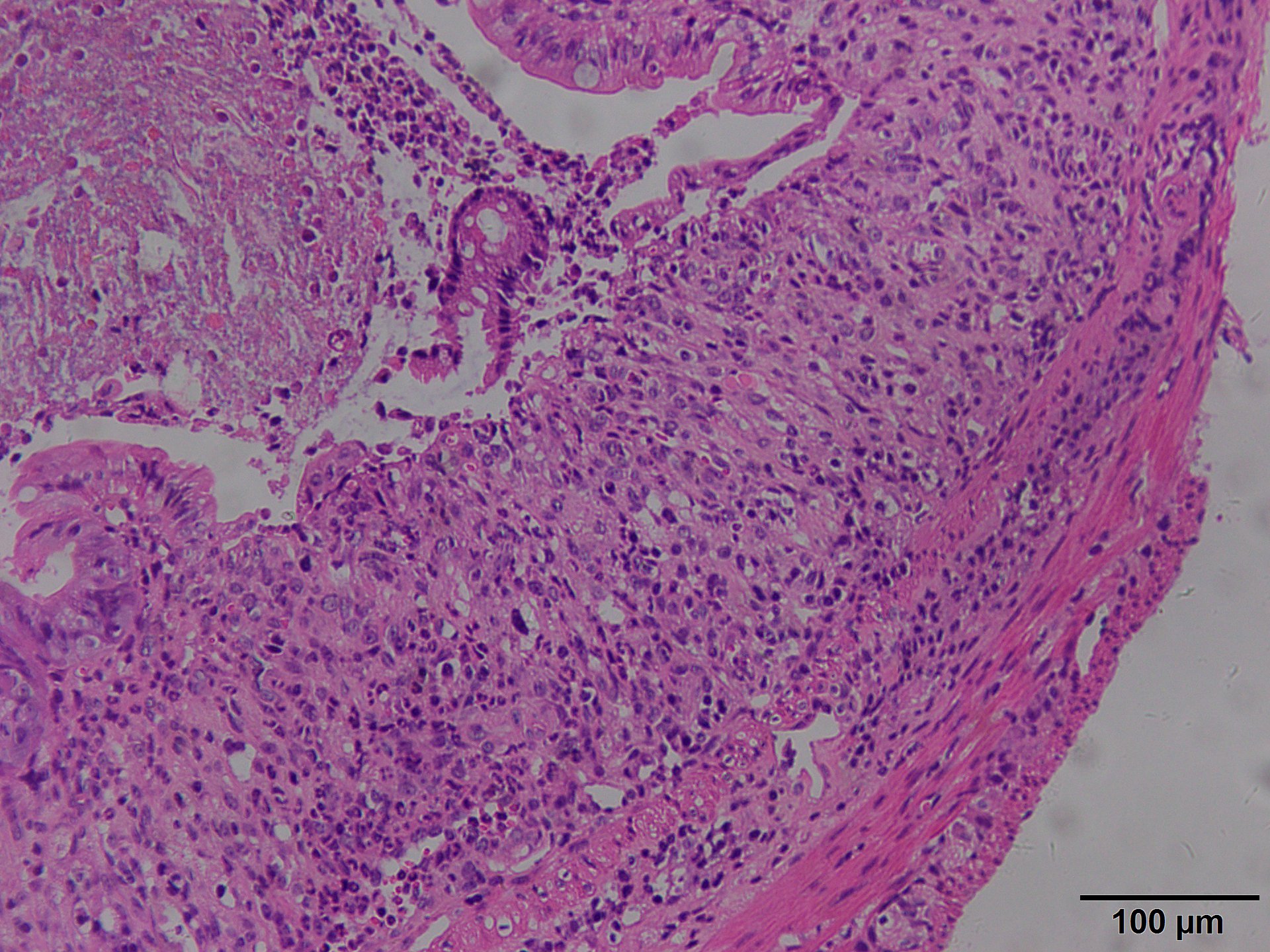

Supplement: Supplementary file 1 [file DataSheet1.ZIP › HE/DSS.jpg]

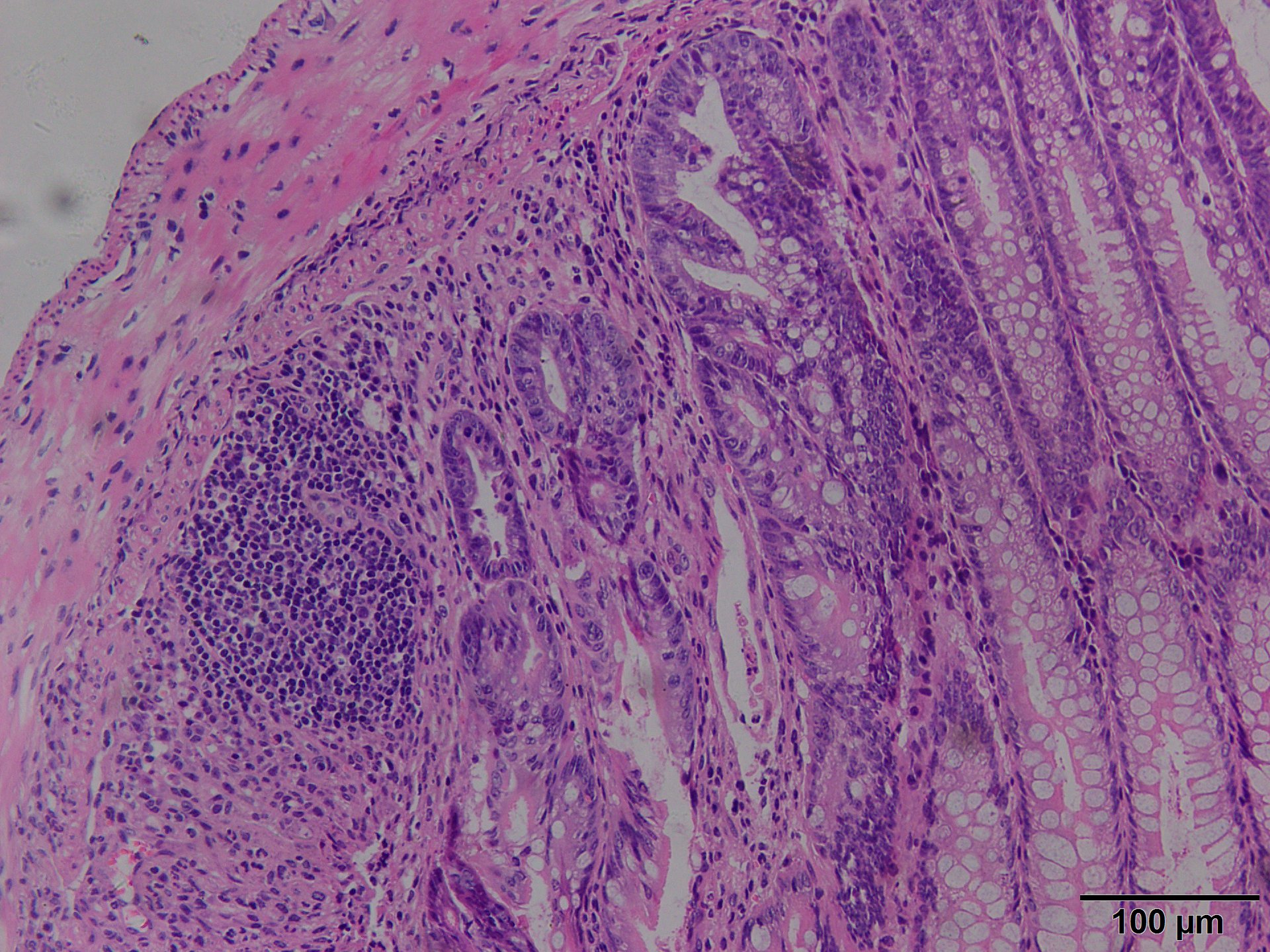

Supplement: Supplementary file 1 [file DataSheet1.ZIP › HE/LB.jpg]

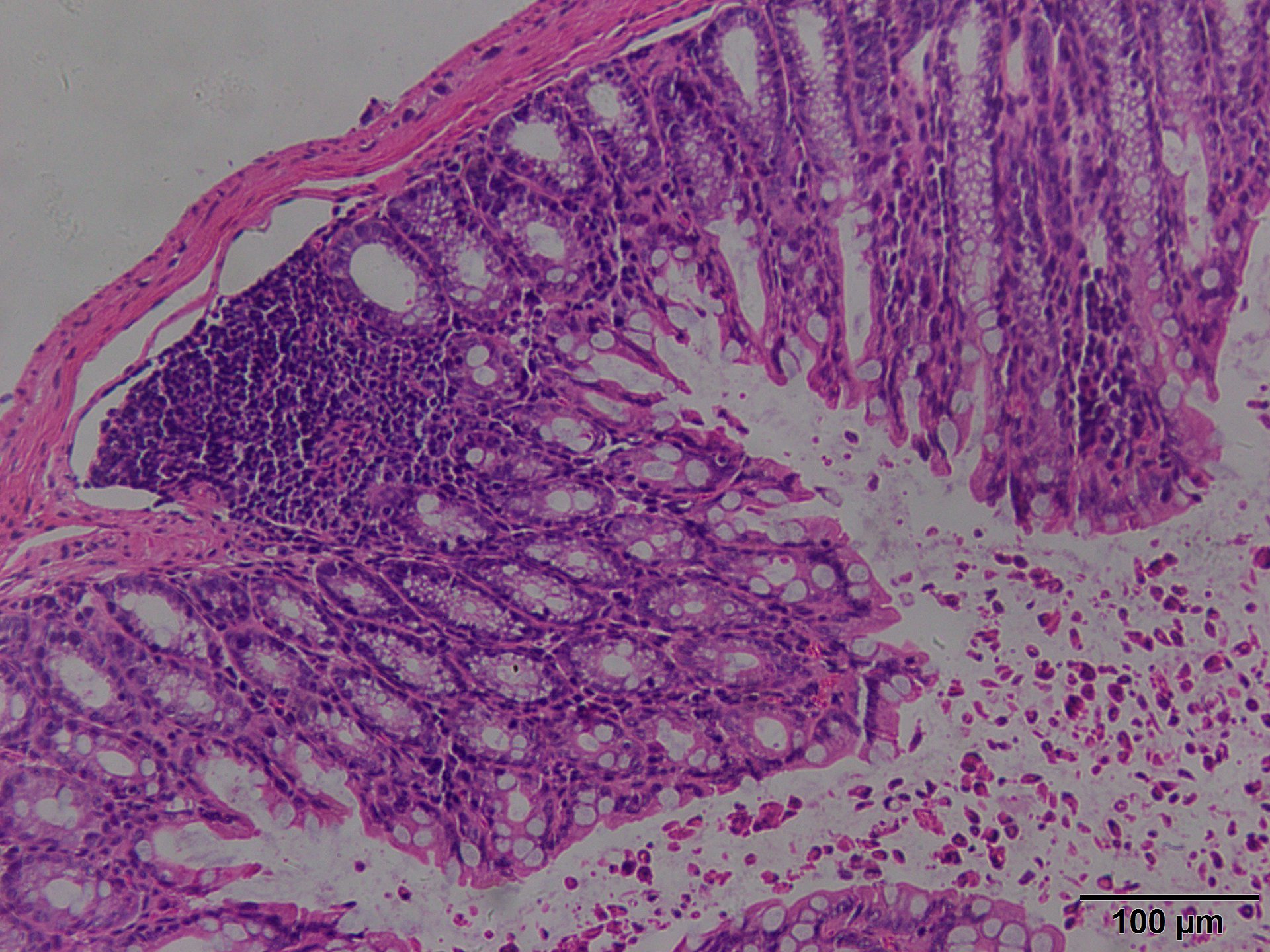

Supplement: Supplementary file 1 [file DataSheet1.ZIP › HE/SSZ.jpg]

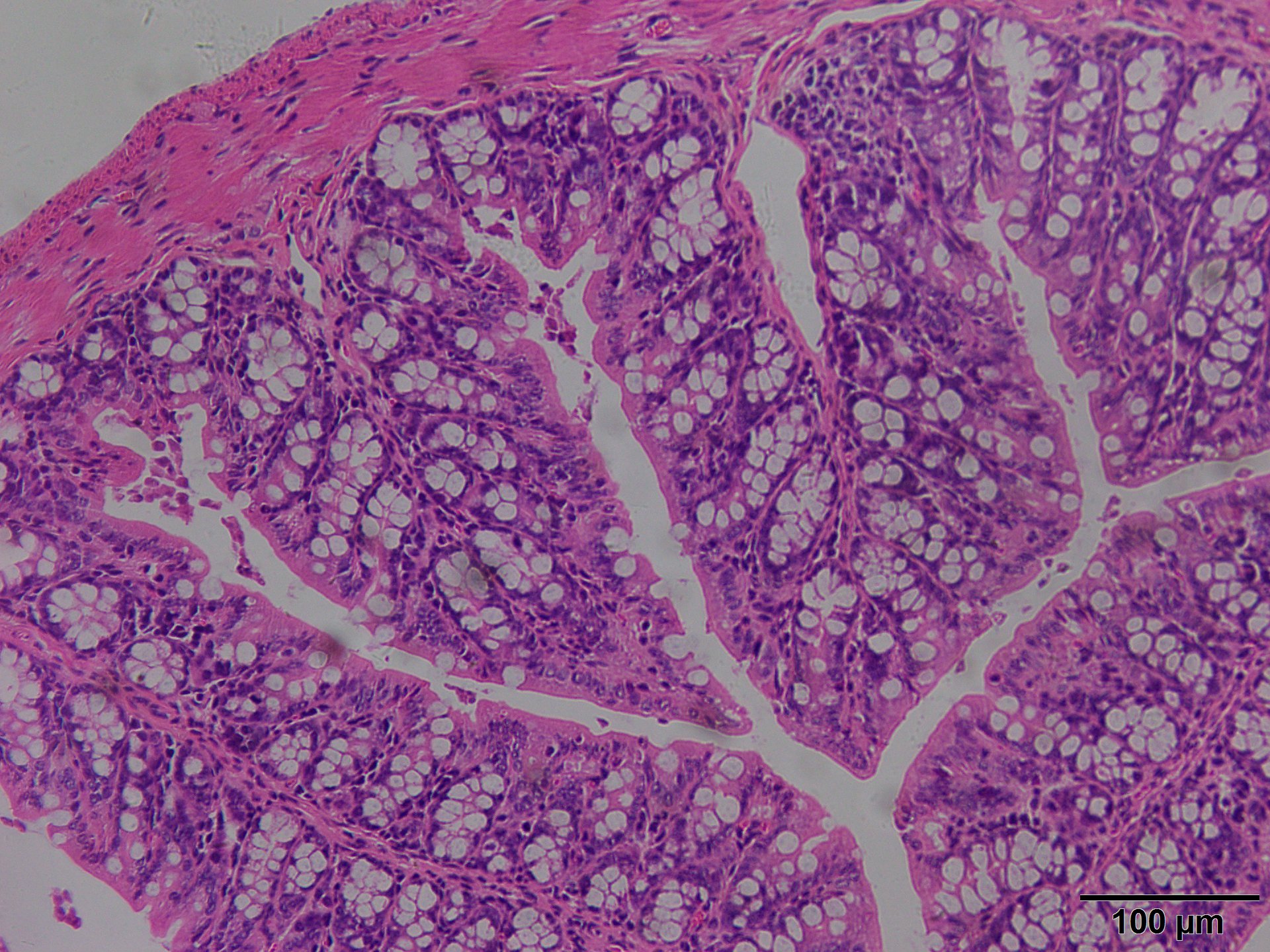

Supplement: Supplementary file 1 [file DataSheet1.ZIP › HE/ZS40.jpg]
